# On the Dark Side of Therapies with Immunoglobulin Concentrates: The Adverse Events

**DOI:** 10.3389/fimmu.2015.00011

**Published:** 2015-02-05

**Authors:** Peter J. Späth, Guido Granata, Fabiola La Marra, Taco W. Kuijpers, Isabella Quinti

**Affiliations:** ^1^Institute of Pharmacology, University of Berne, Berne, Switzerland; ^2^Department of Molecular Medicine, Sapienza University of Rome, Rome, Italy; ^3^Department of Pediatric Hematology, Immunology and Infectious Disease, Academic Medical Centre, University of Amsterdam, Amsterdam, Netherlands

**Keywords:** adverse events, hemolysis, thrombosis, complement, cytokines, SCIG, IVIG

## Abstract

Therapy by human immunoglobulin G (IgG) concentrates is a success story ongoing for decades with an ever increasing demand for this plasma product. The success of IgG concentrates on a clinical level is documented by the slowly increasing number of registered indication and the more rapid increase of the off-label uses, a topic dealt with in another contribution to this special issue of Frontiers in Immunology. A part of the success is the adverse event (AE) profile of IgG concentrates which is, even at life-long need for therapy, excellent. Transmission of pathogens in the last decade could be entirely controlled through the antecedent introduction by authorities of a regulatory network and installing quality standards by the plasma fractionation industry. The cornerstone of the regulatory network is current good manufacturing practice. Non-infectious AEs occur rarely and mainly are mild to moderate. However, in recent times, the increase in frequency of hemolytic and thrombotic AEs raised worrying questions on the possible background for these AEs. Below, we review elements of non-infectious AEs, and particularly focus on hemolysis and thrombosis. We discuss how the introduction of plasma fractionation by ion-exchange chromatography and polishing by immunoaffinity chromatographic steps might alter repertoire of specificities and influence AE profiles and efficacy of IgG concentrates.

## Introduction – The Tinge of the Dark Side of Therapies with Immunoglobulin Concentrates

**Figure 1:**
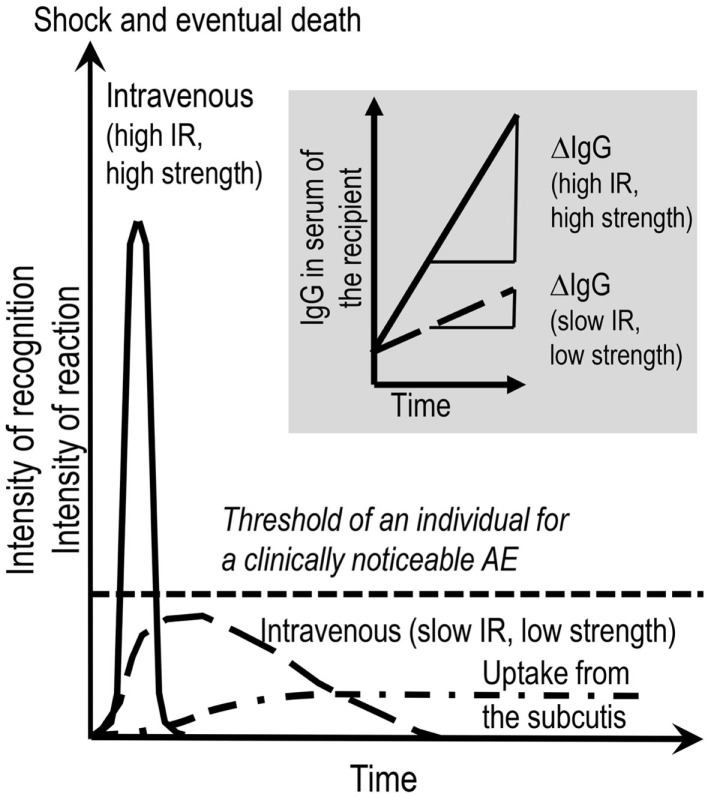
**Provoking the dark**. AEs to therapeutic IgG concentrates might be evoked by several factors. These are listed in the introductory part and Table 1. Contemplating on the active ingredient of the concentrates, the administration of the IgG molecules inevitably results in the interactions of the exogenous IgG with the various parts of the immune system of the recipient and *vice versa*. This interaction in its principle generates an inflammatory condition. A key parameter deciding on the intensity of such a condition is the rate of increase over time of exogenous IgG in the circulation of the recipient. Insert to figure (shadowed): ΔIgG in the circulation over Δtime is dependent on the combination of infusion rate (IR) and the strength of the solution applied. Main figure: the mode of application, i.e., intravenous or subcutaneous, are additional factors being decisive for kinetics and the area under the curve of generation of pro-inflammatory mediators and for passing the individual threshold (short-dashed line) for a clinically noticeable AE. The threshold in turn depends on eventually exiting risk factors form the patient side. Although the area under the curve might be the same for low (long-dashed line) and high (solid line) IRs, at high IR, the system might not be able to cope with the extent of reactions. At low IR (long-dashed line), all the events might remain below the threshold of clinically observable AE. Subcutaneously applied IgG concentrate reaches the circulation slowly and systemic AEs are less frequent than with IVIG. In contrast, local mild to moderate AEs are more frequent with SCIG.

Since the initial clinical use of immunoglobulin G (IgG) concentrates of human origin, transmission of pathogens and non-infectious adverse events (AEs) were reported (1–7). Before the mid 90s, transmission of pathogens depended on the pool size and the fractionation methods used, particularly the polishing steps of an IgG concentrate (8). Mode of fractionation, i.e., cold-ethanol or ion-exchange chromatography, contaminants, route of application, i.e., intra muscular (IMIG), intravenous (IVIG), or subcutaneous (SCIG), the rate of increase of the exogenous IgG in the circulation of the recipient over time and, last but not least an eventually existing risk factor from patients’ side (Figure 1) as well as incorrect handling of the concentrate are factors having a role in inducing non-infectious AEs related to administration of IgG concentrates (Table 1). IgG concentrates represent a defined part of the adaptive immune system, are isolated from pooled human plasma of at least 1000 donors, which contribute to the repertoire diversity in the final product. Therapies with IgG concentrates manufactured according to regulators requirements are acknowledged to be safe in general. This does not exclude the occurrence of AEs which in their majority are rare and clinically mild to moderate. Below, we like to give a few insights into various aspects and possible mechanisms of AEs.

**Table 1:** **The tinge of the dark**.

Symptoms and signs	Frequency	IRR or total dose	PRR	System	Class, severity, and duration	Part of the product likely being involved in AEs
Fatigue	Common (SCIG as well)	No		Constitutional or systemic (generalized)	Immediate, mild, transient	
Malaise	Common	No	Yes	Constitutional or systemic (generalized)	Immediate, mild, transient	
Fever	Common	Yes	Yes	Constitutional or systemic (generalized)	Immediate, mild, transient	
Flushing	Common	Yes	Yes	Constitutional or systemic (generalized)	Immediate, mild, transient	
Chills	Common	Yes	Yes	Constitutional or systemic (generalized)	Immediate, mild, transient	
Anorexia	Common	No		Constitutional or systemic (generalized)	Immediate, mild, transient	
Myalgia	Common	Yes	Yes	Constitutional or systemic (generalized)	Immediate, mild, transient	
Arthralgia	Common	Yes	Yes	Constitutional or systemic (generalized)	Immediate, mild, transient	
Joint swelling	Common	Yes	Yes	Constitutional or systemic (generalized)	Mild, transient	
“Flu-like” symptoms	Common	Yes	Yes	Constitutional or systemic (generalized)	Immediate, mild, transient	Increase in A (dimers)
Anaphylactoid symptoms	RareComplement activationImmune complexes (presence of acute infection)	No	Yes	Constitutional or systemic (generalized)	IgA: acute to immediate; other late, severe transient	I: IgA, very rare immune complexes
Full blown anaphylaxis	Rare Complement activation (in the presence of acute infection)	No	Yes	Constitutional or systemic (generalized)	Late, severe, hopefully transient (ICU)	I: IgA, very rare immune complexes
Headache	Common	Yes	Yes	Neurologic	Immediate, mild, transient	Increase in A
Migraine	Common	Yes	Yes	Neurologic	Transient	Increase in A
Dizziness	Common	Yes	Yes	Neurologic	Transient	Increase in A
Aseptic meningitis	Rare	No	No	Neurologic	Delayed, moderate, transient	Increase in A
Diffuse pain, muscle pain	Rare	Yes	Yes	Neurologic	Transient	Increase in A
Dysesthesia	Rare		Contributes	Neurologic		Increase in A
Weakness	Rare		Contributes	Neurologic		Increase in A
Persistent headache	Rare		Yes	Neurologic	Delayed, moderate	Increase in A
Shortness of breath	Common	Dose	Yes	Respiratory		
Bronchospasm	Common	Yes	Yes	Respiratory		
Pleural effusion	Rare	Dose	Contributes	Respiratory	Severe, transient	
TRALI	Rare	Dose	Likely	Respiratory	Late, severe, transient (ICU)	
Hypotension	Common	Yes	Yes	Cardiovascular	Immediate, mild, transient	
Hypertension	Common	Yes	Contributes	Cardiovascular	Immediate, mild, transient	
Tachycardia	Common	Yes	Yes	Cardiovascular	Immediate, mild, transient	
Chest/back pain	Common	Yes	Yes	Cardiovascular	Immediate, mild, transient	
Arrhythmia	Rare	Dose	Contributes	Cardiovascular	Severe, hopefully transient	
Myocardial infarction	Rare	Dose	Contributes	Cardiovascular	Severe to fatal	Increase in A
Anorexia	Common			Gastrointestinal		
Nausea	Common	Yes	Yes	Gastrointestinal	Immediate, mild, transient	
Vomiting	Common	Yes	Yes	Gastrointestinal	Immediate, mild, transient	
Cramping	Common	Yes	Contributes	Gastrointestinal		
Diarrhea	Common		Contributes	Gastrointestinal		
Colitis	Rare		Contributes	Gastrointestinal	Late, severe	
Tubular swelling	Rare	Dose	Contributes	Renal	Severe, reversible; scars might remain	E: sucrose > > other sugars
Renal failure	Rare		Contributes	Renal	Delayed. severe, ICU	Increase in A (Complement deposition)
Infusion site pain, swelling, erythema	Common (SCIG more frequent)			Cutaneous	Immediate, mild, transient	SCIG: volume
Urticaria	Common		Yes	Cutaneous		Increase in A
Non-specific macular or maculopapular eruptions/eczema	Common		Yes	Cutaneous		Increase in A
Pruritus	Common		Contributes	Cutaneous		
Erythema multiforme	Rare		Contributes	Cutaneous		Increase in A
Cutaneous vasculitis	Rare	Dose	Contributes	Cutaneous	Delayed, severe	
Hemolysis (clinically not significant)	Common	Yes	Contributes	Hematologic	Delayed, moderate, transient	
Acute hemolysis/hemolytic anemia	Rare	Yes	Yes	Hematologic	Delayed, severe	Increase in A
Thrombotic phenomena (DVT, stroke, cardial infarction)	Rare	Yes	Yes	Hematologic	Severe, ICU	Increase in A
Hyperviscosity	Rare	Yes	Contributes	Hematologic	Immediate	Increase in A
Neutropenia	Rare	Yes		Hematologic	Delayed, mild, transient	Increase in A
Blood borne infectious disease	Rare	No	No	Microbiological	Late, severe	I: blood borne viruses, spongiform encephalopathy agent
Inappropriate handling before infusion			No		Immediate, mild to severe	A: incomplete dissolution of lyophilized product; denaturation and aggregate formation due to foam
						E: lyophilized product dissolved to result in too high concentrations; lyophilized product dissolved to result in too high osmolality; low temperature of concentrate at the time of infusion

*The various manifestations of AEs in recipients of polyvalent immunoglobulins. Frequency, severity, duration, timing, possible causes, risk factors*.

*A, active ingredient = IgG; I, impurities; E, excipients/stabilizers; “immediate”, immediate reaction – within 6 h from the onset of infusion; “delayed”, delayed reaction – 6 h to 1 week after infusion; “late”, late reaction: weeks to months after infusion; IRR, infusion rate-related; PRR, patient-related risk factors (acute infection at the time of infusion); ICU, intensive care unit; SCIG, IgG concentrate for subcutaneous application*.

## Pathogen Safety of IgG Concentrates – How to Exclude the Menaces from a Dark and Frightening Environment

**Figure 2:**
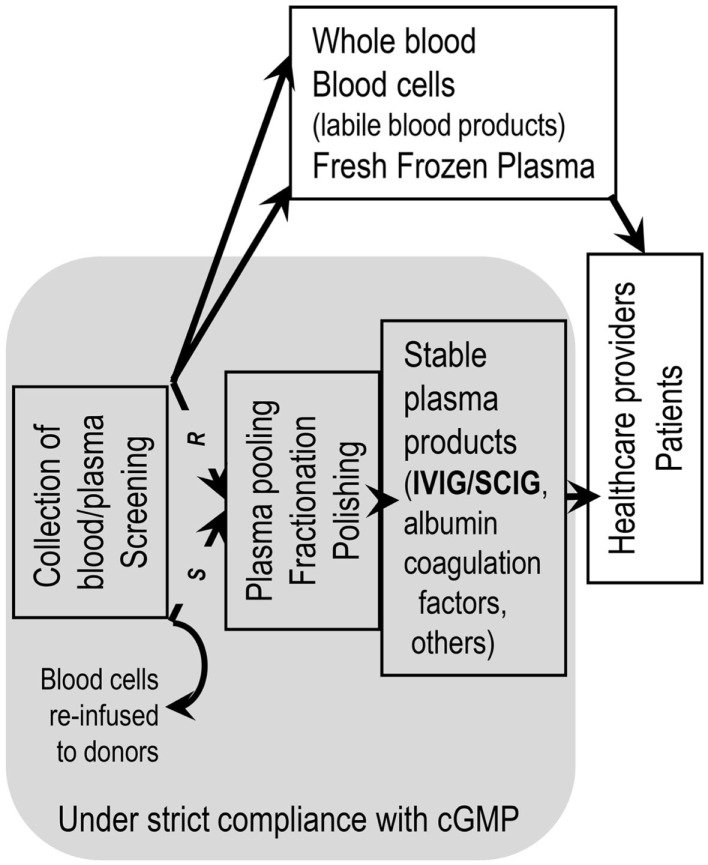
**Staying within a regulatory network is mandatory for remaining at the sunny side of the moon**. Handling of blood/plasma products to obtain therapeutic goods has to be performed within a regulatory framework. Manufacturing of stable blood product has to adhere strictly to current good manufacturing practice (cGMP). Application of cGMP starts from the moment of collecting whole blood and isolation of plasma thereof (R = recovered plasma) or the machine-supported collection of plasma (S = source plasma, apheresis plasma). Application of cGMP ends at delivery of blood/plasma products to health care professionals. Not following cGMP can lead to withdrawal of a plasma product from the market from one to the other day.

**Figure 3:**
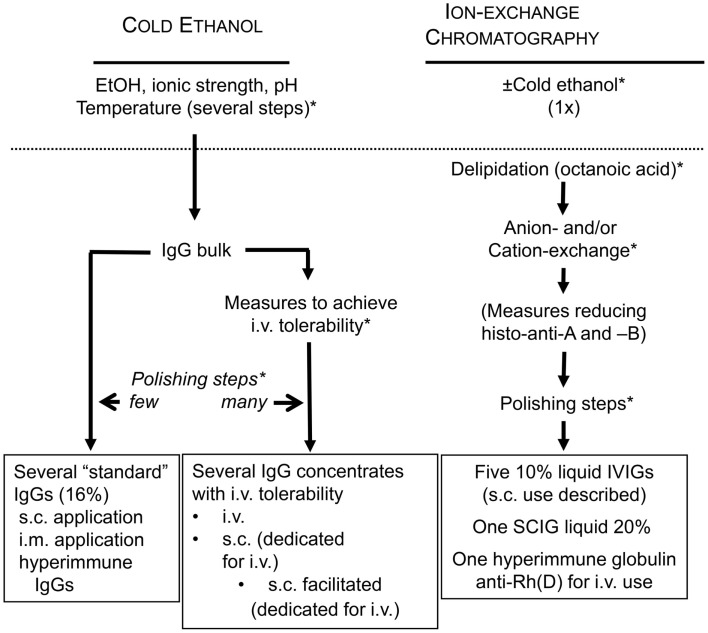
**Active measures in fractionation to stay on the sunny side of the moon**. (*) depicts possible sites for validating the efficiency of already established fractionation steps or for introducing dedicated pathogen reduction and/or inactivation measures. Histo-blood group antibodies were made responsible for hemolytic AEs. Reduction of these during the manufacturing process is possible by introduction of an immunoaffinity chromatographic step in order to reduce anti-A and anti-B titers.

Manufacturing of modern IgG concentrates has to occur in a regulatory framework and the quality standards implemented by the plasma fractionating industry (Figure 2). The cornerstone of the regulatory framework is current good manufacturing practice (cGMP). A pillar of pathogen safety is the validation of virus inactivation and virus elimination methods by validating an already performed step of the fractionation process or by introduction of dedicated steps (Figure 3). A hallmark of virus elimination introduced in the late 90s in Berne by the team of Christoph Kempf is the large-scale virus filtration technique (formerly also termed “nanofiltration”) (8). Meanwhile, virus filtration became a versatile tool to eliminate a variety of pathogens, the suspected agent of variant Creutzfeldt-Jakob disease included. Thanks to the tightly implemented regulatory framework, pathogen safety of plasma products is at a level never reached before. This is well supported by the fact of reports missing in the last decade of transmission by IgG concentrates of emerging viruses (SARS coronavirus, West Nile Virus, MERS coronavirus, and others), zoonotic pathogens, or the agent of variant Creutzfeldt-Jakob disease (vCJD). Furthermore, the development of specific mass screening techniques might help to eradicate in any blood product the transmission of vCJD in the future (9).

## How the Shadow Might Grow – Some Basic Insights into Possible Mechanisms of Non-Infectious Adverse Events

The human immune system is in charge of controlling invading organisms and mediates homeostasis. The immunoglobulin pools in mammals (IgM, IgG, and IgA) to its smaller part provide defense and to the larger part homeostasis. Human IgG has a role in both. Efficient host defense is supported by “immune antibodies.” These have undergone somatic hypermutations and have in their vast majority narrow specificities and high affinities. Antibodies, generated in absence of external stimuli are termed “natural antibodies” (NAbs). They occasionally recognize self structures. In general, the V(D)J genes of NAbs are in germ-line configuration or have undergone a few somatic hypermutations only. Furthermore, they have broad specificity, are of low affinity (with exceptions) and high avidity (10). These NAbs can participate in primary host defense, i.e., at a time point when an immunologic reaction has not provided the specific antibodies, react, e.g., with repetitive structures which can be found on bacteria or viruses (11). The self-reactive NAbs, which we like to term physiologic autoantibodies, comprises various populations of antibodies such as (i) those able to interact through their complementary variable regions (V-regions) with the V-regions of circulating and membrane-bound (BCR) immunoglobulins and the T cell receptor (TCR) β-chain variable region, providing a peripheral immune network (V-connected network) (10); (ii) the NAbs reacting in a non-idiotypic manner with the hinge region of immunoglobulins (12, 13); (iii) populations of NAbs showing a wide variety of specificities toward growth factors, cytokines, or anaphylatoxin (10); (iii) and the population reacting with the soluble or membrane-bound forms of cell surface molecules having immunological importance, the last described being the Fc receptors CD16 and CD32 (14). Antibodies reacting with docking structures for viruses or bacteria can have additional first-line defense potential (15). These populations of NAbs were described having a peripheral immune network homeostatic and anti-inflammatory function (16, 17). Although the primordial humoral proteins comprising the complement and lectin-like proteins in the plasma play a definite role, another population of self-reactive NAbs reacting with, e.g., epitopes conserved over the evolution apparently has tissue homeostatic function and might support the efficient removal of roughly 10^12^ altered/senescent cells of the body per day (for references see below). The signal for research on NAbs in IVIG was the description of IgG autoantibody-mediated immune thrombocytopenia (ITP) being corrected by infusion of a polyclonal, polyspecific IgG concentrate (18, 19). This research has expanded ever since.

The populations of immune antibodies and NAbs in IgG concentrates upon infusion/injection inevitably react with occasional pathogens, toxins, or superantigens and concomitantly infusion/injection also results in recognition of a wide array of tissue antigens and V-regions of the recipient’s immune system. Reactions with tissue antigens and V-regions are conveyed by the self-reactive antibodies of the many donors in the IgG concentrate. *Vice versa*, the recipient’s immune system reacts with the infused IgG. A bewildering wide range of possible reactions can occur which primarily are dependent on the immune status of the recipient at the time of therapy and to a smaller part on the IgG concentrate(s). The therapeutic effect achieved depends on the disease treated, and can depend on the concentration reached locally, i.e., can have agonistic or antagonistic effects (17, 20–22). In summary, it is our opinion that IgG concentrates always provide more or less the same “bouquet” of IgG specificities (similarity); however, it is the recipient’s actual immune condition which decides from which IgG specificities the patient’s derailed immune system is profiting (diversity).

Parameters of IgG-mediated AEs are: (i) the content in the product of biologically highly active likely beneficial ingredients that have to be kept under control (e.g., content of “dimers” devoid of remarkable complement activation *in vivo*; see below), and the content in unwanted active ingredients that have to be discarded during manufacturing (alloantibodies); (ii) impurities such as IgA (anaphylactoid reaction); (iii) activated coagulation and contact activation factors (thromboembolic events) and; (iv) excipients such as sucrose (osmotic nephrosis). Below, we like to add and contemplate on how fully native IgG molecules not harmed by the manufacturing process might add to AEs. The above mentioned inevitable interaction of the exogenous IgG with the immune system of the recipient and *vice versa* in its principle might evoke an inflammatory condition. The sum of the potentially beneficial reactions might overshoot and lead to AEs (Figure 1). The principle of induction of mild inflammatory conditions upon each infusion of a well-tolerated IVIG was confirmed when several dozen normogammaglobulinemic volunteers in all cases except one, showed a more or less moderate inflammatory reaction as indicated by the increase of tumor necrosis factor alpha (TNFα) at 2.5 h post initiation of infusion. The only person in the cohort not showing a measurable TNFα increase was a woman caring at home for her brother with full blown AIDS (22).

Subcutaneously applied IgG concentrate reaches the circulation slowly and systemic AEs are less frequent compared to IVIG but they are not absent (23–28) (a case of unintentional i.v. application of SCIG is not considered). In contrast, local mild to moderate AEs are more frequent with SCIG (29). In summary, the intensity of the resulting AEs is depending on the immune status of the recipient, the infusion rate (IR), e.g., how rapidly the active ingredients (the various IgG specificities), the impurities, and the excipients reach the circulation of the recipient. Thus, the i.v. application has the highest chance for the occurrence of AEs.

In the early days of IVIG therapy, complement-mediated “anaphylactoid” (i.e., immediate) and “phlogistic” (i.e., inflammatory) AEs were distinguished (30, 31). The complement-mediated AEs were considered to be caused by aggregates in the product (“spontaneous complement activation” or anti-complementary activity or ACA) or by *in vivo* formation of immune complexes (ICs, patient’s condition related; e.g., subclinical infections or the unnoticed presence of anti-IgA antibodies) and therefore only IgG concentrates with low or absent ACA is accepted by authorities for human use. Below, we present one instructive case of each type of reaction.

### Immediate adverse events – the rapid onset of darkness

The first reports of rapid onset AEs concerned either the application of complement-activating fractions in an IgG concentrate or the *in vivo* formation of complement-activating ICs (2–4). A very rare but potentially fatal condition is the formation of IgA/anti-IgA complexes in patients being initiated on replacement therapy and having serum IgG antibodies against infused IgA not recognized before the start of the IVIG infusion (32). Prerequisite for the presence of anti-IgA antibodies is the most common primary immunoglobulin defect, i.e., selective IgA deficiency (sIgAD) or IgAD associated with diminution of other immunoglobulin classes. IgAD is defined by serum levels of <0.05 or <0.07 g/L (depending on laboratories). A marked diminution of serum IgA consistent with IgAD in various ethnic groups is estimated being 1:155 to 1:18,550 (33). The mean frequency in Caucasians is approximately 1:700 (34). Up to 40% of patients with IgAD have been reported having anti-IgA antibodies in the serum with titers ranging between 1:4 and 1:262,144. In approximately 10% of patients with common variable immunodeficiency (CVID), and occasionally in patients with other primary immunodeficiency diseases, measurable anti-IgA can be detected (35, 36). These antibodies are predominantly of the IgG class, but anti-IgA antibodies of other immunoglobulin classes have been described as well (37, 38). The reason for their emergence remains unknown.

Taken the above numbers, the infusion of human-derived products containing IgA resulting in severe anaphylactoid type AEs should be considerable. This is not the case (39). Questions about the clinical relevance of above numbers emerge as soon as blood banks (i) estimate the theoretical risk of IgA anaphylactic reactions (32); (ii) assess the relation of severe IgAD with the presence or absence of anti-IgA antibodies (40); (iii) screen donors for very low IgA levels in order to become able to provide blood and plasma-derived products free of IgA and find a considerably lower frequency than expected (41). Alternatively, the test systems may not reliably detect anti-IgA antibodies being as yet insensitive and inaccurate or – at least – do not correspond to the clinically relevant fraction of antibodies. This comes to mind when a more close look to “anti-IgA” gives “unexpected” results, including “anti-IgA” in blood donors with normal serum IgA level or “anti-IgA” that cannot be neutralized with purified IgA (42); or when blood products containing proven anti-IgA do not elicit severe AEs (43).

Among patients on replacement therapy, those with CVID may rarely develop severe immediate AEs (32). The discrepancy between anti-IgA positive patients and frequency of AEs raises the question about the nature of the many reported anti-IgA antibodies and also raises the question about the immunologic condition which allows the formation of anaphylactoid anti-IgA antibodies. There might be some logic in supposing that anaphylactoid anti-IgA cannot evolve at IgA levels otherwise fulfilling the definition of IgAD. Such a condition would constantly generate ICs which in turn could activate complement, react with immune cells, and be deposited in lung and kidney. Indeed, Horn et al. found anti-IgA antibodies in CVID patients missing IgA^+^ B cells and presenting with IgA levels <0.0009 g/L, a level which is more than 50- to 70-fold lower than the threshold for IgAD (44). However, a possibility for an IgA-mediated anaphylactoid reaction at measurable IgA serum levels might exist. Serum IgA contains approximately 85% subclass 1 of IgA (IgA1) and only 15% subclass 2 of IgA (IgA2). Selective deficiency of IgA2 and – although evidence is lacking – the presence of a highly specific anti-IgA2 antibody theoretically could elicit a severe AE.

**Figure 4:**
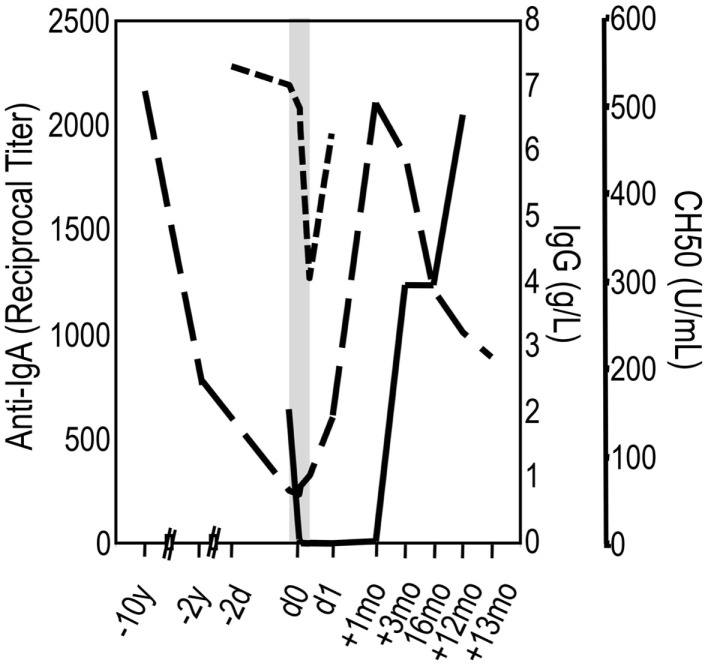
**An individual’s slithering into the dark**. A female patient has been suffering from recurrent airway infections since adolescence, occasionally complicated by pneumonia. At age 34, selective IgA deficiency was diagnosed. Ten years later, she was hospitalized with pneumonia. Within these 10 years, her serum IgG had dropped from 7 to 0.87 g/L (long-dashed line) and IgA was undetectable. The diagnosis was corrected into CVID, and IVIG replacement therapy was initiated (shadowed area). Two minutes after the start of the infusion of a 3% IgG solution (IgA < 1.2 g/L; 3% solution), she experienced a flush, back pain, rigors, difficulty in breathing, and hypotension. The infusion was immediately stopped and later continued without further complications (see text). The confirmation of a true anaphylactoid reaction due to anti-IgA in the serum of the patient was achieved by follow-up of anti-IgA (solid line) and CH50 (short-dashed line).

The kinetics of anti-IgA after infusion of blood products have been studied in a few cases. In these patients, a fall in anti-IgA titers has been noticed followed by an increase during subsequent weeks or months. This suggests that at appropriate proportions, IgA of the infused material and anti-IgA present in the patients’ serum combine with each other to form ICs. In turn, ICs activate complement that are bound and eliminated by macrophages most likely leading to cytokine release. The increase in anti-IgA titers over time indicates that the infused IgA-containing product has a booster effect (36, 37, 45). Such boosting effect together with the presence of anti-IgA before the application of an IgG concentrate can be taken as the ultimate confirmation of a supposed IgA/anti-IgA reaction. Figure 4 depicts a well-documented case of IgA/anti-IgA reaction in a patient who progressed from sIgAD to CVID. The events during the first 12 h at occasion of the first infusion of IVIG were as follows (shadowed area in Figure 4): 2 min after the start of the infusion, having received eight drops of an IgG solution (IgA < 1.2 g/L; 3% solution), she experienced a flush, back pain, rigors, difficulty in breathing, and hypotension. The infusion was immediately stopped. After approximately 1 h, the reaction has weaned, and 2 h later the patient felt well again, and the infusion of total 6 g IgG could be continued without further complications. Although the patient fairly assured having never received any blood or plasma product in the past, the follow-up of her anti-IgA titers from before infusion to 1 year later confirmed a true anaphylactoid reaction mediated by anti-IgA, as the anti-IgA became undetectable immediately after the infusion and showed a boosting phenomenon during the following months. True anaphylactoid reaction was further confirmed by follow-up of total complement hemolytic activity (CH50) on the day of infusion. Interestingly enough, the CH50 value reached its nadir at the end of the infusion when the patient had no complains. Although a single case only, the events during the first infusion call for the following remarks: (i) severe AEs most likely occur at concomitant complement and cell activation with cytokine release; (ii) infusion of minute to low amounts of IVIG hours before the main infusion can “anergize” cells and stop release of pro-inflammatory cytokines; (iii) “anergized” cells loose reactivity toward ongoing formation of ICs and complement activation products.

A non-complement-mediated anaphylactoid reaction was ascribed to the unforeseen release of elastase and other pro-inflammatory substances from neutrophils activated by the formation of *in vivo* IgA/anti-IgA complexes. Complement activation or mast cell-dependent release of vasoactive substances was excluded as pathogenic mechanisms. Although the IgA/anti-IgA complexes usually do not cause clinically relevant neutrophil degranulation within the circulation, the presence of a rare genotype encoding a novel gain-of-function IgG receptor on neutrophils may provoke premature degranulation by these complexes. This phenomenon was only relevant in hypogammaglobulinemic patients in the presence of *in vivo* IgA/anti-IgA complexes (46). The low prevalence of this genotype combined with an IgAD or CVID may add how to explain the rarity of serious anaphylactoid reactions in newly IVIG-treated patients. Authors share the opinion of Janne Björkander who at occasion of a discussion panel “Dilemmas in Diagnosis and Management of Antibody Deficiencies: Ask the Experts” held at occasion of the 58th Annual Meeting of the American Academy of Allergy, Asthma & Immunology (AAAAI), New York City, March 1–6, 2002 came to the following conclusion: a clinician has to be aware of the risk, particularly at occasion of first infusions, but otherwise IgA is not a major concern (from tape record).

### Pro-inflammatory cytokines – the phlogiston of the dark

**Figure 5:**
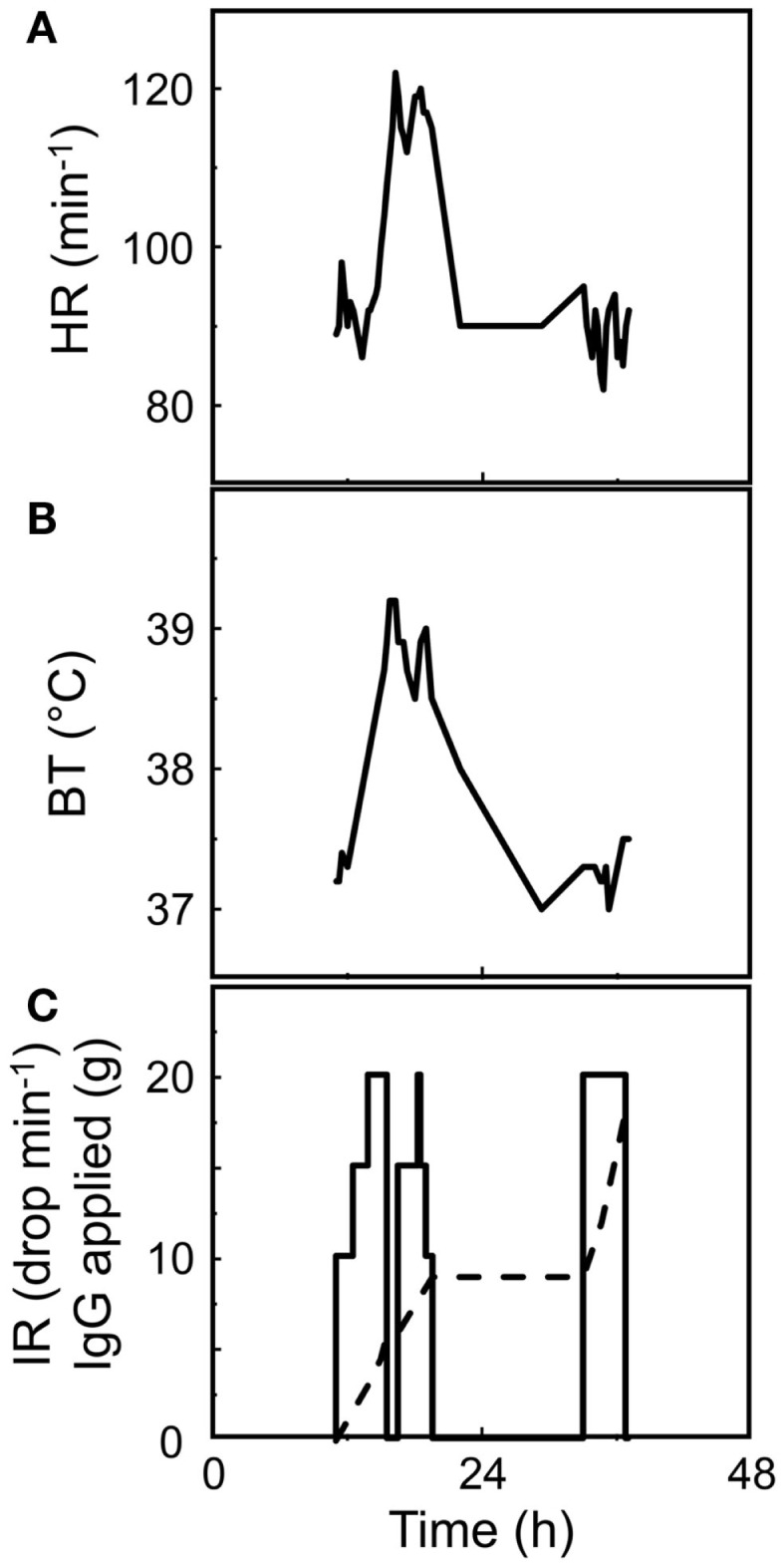
**Clinical signs of the “phlogiston” of the dark**. A CVID patient received his first infusion of IVIG (a 3% solution). Despite the start of the infusion at an infusion rate [IR, **(C)**] of only 10 drops per minute and incremental increase by five drops every 30 min, rise of heart rate [HR, **(A)**], and of body temperature [BT, **(B)**] after 2 h of the initiation of infusion indicated the onset of a “phlogistic” reaction and infusion was stopped. Stop of the infusion for 30 min immediately let drop HR and BT. Restarting the infusion showed some negative effect and infusion was stopped after application of 9 g IgG [dashed line, **(C)**]. The next day the rapid infusion of additional 9 g of IgG was without consequence on BT and HR, indicating silencing of cells releasing mediators of inflammation.

In the early days of Ig-therapy, the nature of the “phlogistic” AEs was obscure. However, it was already known that an AE can be prevented or its evolution halted when the patient receives a low dose of IVIG first or the infusion is stopped early and is continued several hours later. Hours later the infusion can be (re)started at high rates without further problems (Figure 5). One of the authors had a particular opportunity to get an insight into what a “phlogistic reaction” might be. At the occasion of a voluntary infusion of an investigational liquid IVIG, he encountered a severe flu-like AE of more than 12 h duration. Before injection, the investigational liquid preparation had passed all release criteria for human use, including spontaneous complement activation assessed by ACA and was free of prekallikrein activator (PKA). In those days, assays for cytokines in biological samples just began to become available and were included into the parameters assessed in the study. Infusion was stopped after 1 h because of a drop of pulse rate and heavy discomfort provoking the laconic comment by the proband’s technician who was taking samples: “you look green.” The infusion was continued after another 90 min when the heart rate had almost normalized. The infusion could be completed within an additional 3.25 h (a total of 0.4 g/kg b.w.) without further aggravation of malaise. The leukocyte count transiently had dropped to a nadir of 40% at 2 h followed by a leukocytosis peak at 8 h. Complement activation, as assessed by generation of C3a/C3a[desArg] and the formation of the terminal complement complex C5b-9, apparently did not occur: the C3a/C3a[desArg] value reached a maximum of 260 ng/mL (norm: <200 ng/mL) at 7 h while the C5b-9 value never moved outside the normal range. Instead, a sequence of rapid transient massive increases of pro-inflammatory cytokines was observed: (i) TNFα started to increase 30 min post initiation of infusion from a value of 20 pg/mL to a peak value at 2 h which was above the calibration range of the test kit of 1500 pg/mL; (ii) interleukin 8 (IL-8) increase started after 1 h from 29 pg/mL and peaked at 2.5 h with 4400 pg/mL post initiation of infusion; (iii) interleukin 6 (IL-6) secretion started after 1 h with an undetectable level and peaked at 4 h with 345 pg/mL. All pro-inflammatory cytokines fell sharply while the second part of the infusion was still ongoing. The day after infusion, the pro-inflammatory cytokine profiles were back to normal and the flu-like syndrome was gone. In contrast, interleukin 1 receptor antagonist (IL-1ra) values started increasing at 1.5 h (300 pg/mL), peaked at 4 h (>32,000 pg/mL), and decreased slowly to reach a value of 10,500 pg/mL 24 h after initiation of the infusion. Soluble TNF receptor p75 level started at 2.25 ng/mL, reached a peak with 16.5 ng/mL at the same time as IL-1ra, and 24 h after initiation of infusion was still at 10.5 ng/mL. Thus, in this normogammaglobulinemic subject similar cytokine profiles and leukocyte number changes were observed as reported for hypogammagobulinemia under replacement therapy (47, 48).

A series of further experiments with investigational and marketed IVIGs was performed. All IgG concentrates were analyzed for their molecular weight (MW) distribution. The most remarkable differences emerged in the MW range of dimers while the presence of minute amounts of higher oligomers could not be excluded with certainty. Below, we will use the term “dimers” for that fraction of IgG with higher MW. Subsequent findings indicated that levels of “dimers” >12% were responsible for complement-independent cell activation and cytokine release. The TNFα peaks assessed at 2.5 h post initiation of infusions correlated with “dimer” content of the IVIGs and mirrored a clinical score of AEs (49–51).

A few years before a complement-independent induction of a hypotensive factor by IgG di- and oligomers was reported in animal experiments (52). A key role for macrophages in the generation of the hypotensive lipid factor was identified as platelet-activating factor, being induced by dimers and polymers (53, 54). Several years later, the dimer-mediated AEs in animal experiments were confirmed (55, 56). Yet, at the same time, the dimer content of IVIG apparently correlated with the clinical efficacy in a murine ITP model (55, 57). Variables such as “IR,” “genetic background,” “endogenous immunoglobulin levels,” or “proportional fraction of polymers versus dimers” may impact on the balance between the phlogiston (being cytokines, active lipid substances, or a combination of factors) and the therapeutic efficacy (blocking IgG receptors on liver/spleen macrophages to prevent clearance of “opsonized” material such as platelets in ITP). As of today, reports on release of cytokines in humans in association with AEs or tolerability toward dimers remain scarce and to the best of our knowledge studies in humans of causative factors/fraction in an IgG concentrate has not been adequately addressed (47, 48, 58–61).

In IgG preparations, various forms of dimers might be present: formed through covalent binding (62) by denaturation, hydrophobic interactions of the Fc-parts, and by idiotype/anti-idiotype interactions, as part of the V-connected network of peripheral immune homeostasis (63). For a commercially viable fractionation process, pooling of donated plasma is mandatory in order to obtain a volume of starting material large enough to cover ever increasing costs for documentation, in-process, and batch-release testing as it is required by cGMP. Pooling also intends smoothing the batch-to-batch differences in antibody titers, a goal apparently difficult to achieve to levels as theory might imply (64). Consequences of pooling are on the one hand the enrichment of public/common immune antibodies while diluting out individual specificities; on the other hand, the antibodies of the immune network of an individual donor are exposed to those of many other donors. The more individuals contribute to the pool, the more complex the possible “immune-network” interactions among IgA, IgG, and IgM will become. The subsequent fractionation process has far-reaching effects on immunoglobulins from a given pool: only trace amounts of IgA and IgM are retained in the final product, i.e., IgG is deprived of its counterparts of the V-connected immune network. The IgG molecules of the homeostatic network “naked” at their V-regions can interact with each other at random combining site-interactions of single donor-derived monomeric IgG (65), otherwise not existing *in vivo*. This interaction is largely reversible. With increasing numbers of donors included into the pool, the immune network recognition among the “naked” IgG molecules of the V-connected network becomes more and more complex, and the dimer and lower oligomer content in the resulting IgG concentrate increases (66–68). In lyophilized IgG concentrates, the dimer formation is “frozen” at a low level while in liquid preparations an equilibrium between monomers and dimers is achieved over time reaching a dimer content of 12% or more if not hampered by stabilizers. Specificities, as far as they have been addressed, in the dimer fraction considerably differ from the monomeric fraction (69–73). In conclusion, the immunomodulatory efficacy of IgG concentrates in part depends on the capacity and extent to form “dimer” fractions devoid of remarkable complement activation *in vivo*. The “art” of manufacturing a liquid IgG concentrate is not to eliminate the monomeric IgG having potential for “dimer” formation but to inhibit extensive “dimerization.” In summary, AEs might be associated with the induction of pro-inflammatory cytokines in absence of measurable complement activation *in vivo* where all regulatory mechanisms and removal processes of a body are at disposition. At reasonable IRs in the open system of the human body, clinically relevant systemic complement activation apparently needs oligomers formed of three or more IgG molecules.

## The Missing Oxygen on the Dark Side – Immunoglobulin-Induced Hemolysis

**Figure 6:**
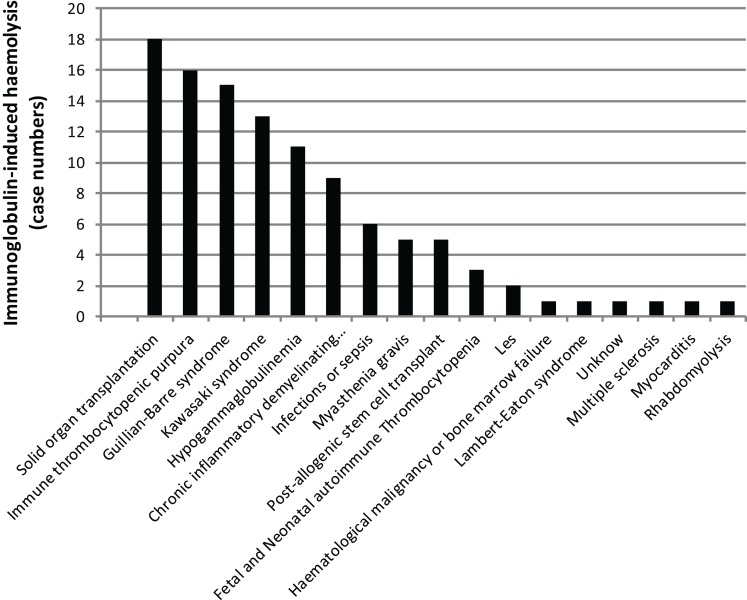
**The missing oxygen on the dark side: Ig-induced hemolysis in patients on Ig treatment**. Black bars indicate the number of patients with a given clinical condition.

There are multiple reports of Ig-induced hemolytic anemia (HA) in patients receiving high doses of IVIG (60, 74–110) (Table 2; Figure 6; www.adrreports.eu). By spontaneous reporting, risk factors recognized for Ig-induced hemolysis include beside high doses (more than 100 g IVIG over 2–4 days), female gender and histo-blood group type A, B, or AB of recipients.

**Table 2:** **The missing oxygen on the dark side – Ig-induced hemolysis in recipients of polyvalent immunoglobulins**.

Publication	Number of patients	Blood group	Monthly Ig dosage (mg/kg)	DAT	Eluted antibody	Alloantibody passively administered	Hemoglobin drop (g/L)	Outcome
Quinti et al. (110)	8	A+ (5), A- (1), O+ (2)	Low	IgG (2), IgG and C3d (4)	anti-A (5), anti-C (1), anti-C and anti-D (1)	anti-A, anti-C, anti-D	6.4, 1.5, 5.1, 1.4, 6.9, 1.1, 1, 1	Recovery (7), death (1)
Desbourouh et al. (76)	1	AB+	High	IgG and C3	anti-A and anti-B	anti-A and anti-B and anti-D	6.5	Recovery
Mohamed et al. (77)	1	A+	High	IgG	anti-A	nd	4	Recovery
Rink et al. (78)	3	A+ (2)	High	IgG	nd	nd	1.2, 3.8, 4.4	Recovery
Berard et al. (79)	4	A+ (2), B+ (1), AB+ (1)	High	IgG	anti-A (1), anti-B (1), anti-A and anti- B (1)	nd	2.9, 5.8, 5.8, 3.7	Recovery
Michelis et al. (60)	1	A+	High	IgG	anti-A	nd	3.5	Recovery
Pintova et al. (80)	2	AB+, A+	Low	IgG	anti-A	nd	6.6, 7.2	Recovery
Morgan et al. (81)	3	AB+(1), A- (1), A- (1)	High	IgG	anti-A (2), anti-A and anti-B (1)	anti-A (2), anti-A and anti-B (1)	4.8, 5.0, 1.8	Recovery
Welles et al. (82)	1	nd	High	IgG	nd	nd	4.3	Death
Canadian Group (83)	20	A (14), AB (6)	High	IgG	nd	nd	3.2, 2.8, 5.1, 5, 5.6, 5, 3.5, 4.1, 7, 3.2, 5.6, 3.2, 6.6, 2.9, 3.1, 4, 3.9, 7.8, 4.9, 4.8	Recovery (10), death (1), unknown (8)
Gordon et al. (84)	4	A+ (3), AB+ (1)	High	IgG	nd	nd	5.3, 5.5, 4.8, 4.8	Recovery
Kahwaji et al. (85)	16	A+ (10), A- (2), B+ (3), AB+ (1)	High	IgG	nd	anti-A, anti-B	5.3, 4.7, 5.6, 4.9, 5.8, 5.7, 3.3, 2.4, 3.1, 4.0, 3.6, 2.1, 2.2, 2.8, 5.3, 1.9, 2.6, 3.0	Recovery
Daw et al. (86)	16	A+ (7), AB+ (1), AB- (1), B+ (6), O- (1)	High	IgG	anti-A (6), anti-B (4)	anti-A, anti-B	1.4, 3.6, 4.3, 3.6, 3.2, 3.4, 3, 4.7, 5.1, 5, 2.4, 8, 5.2, 1.3, 3	nd
Yin et al. (87)	1	AB+	High	Negative	nd	anti-A and anti-B and anti-D	nd	Recovery
Coghil et al. (88)	1	A+	High	IgG	anti-A	anti-A	4	Recovery
Chamouni et al. (90)	1	AB+	High	IgG	nd	nd	8	Recovery
Karaaslan et al. (91)	1	nd	High	IgG	nd	nd	3.9	Recovery
Trifa et al. (92)	1	AB+	High	IgG	anti-A and anti-B	anti-A and anti-B	7.8	Recovery
Nagakawa et al. (94)	1	A+	High	IgG	nd	nd	2	Recovery
Wilson et al. (95)	12	A+ (11), O+ (1)	High (10), low (2)	IgG	anti-A (9), anti-A and anti-D (2), anti D (1)	nd	3.7, 3.8, 1, 1.6, 1.9, 2.9, 1.4, 1.9, 3, 1.8, 1, 0.9	nd
Tamada et al. (97)	2	nd	High	IgG	anti-A, anti-B	nd	nd	nd
Thomas et al. (99)	1	A+	High	IgG	anti-A	anti-A	6.3	Recovery
Comenzo et al. (100)	1	nd	High	nd	nd	nd	nd	Recovery
Okubo et al. (102)	1	A+	High	IgG	anti-A	anti-A	nd	nd
Hillyer et al. (103)	1	AB+	High	IgG	anti-A, anti-B	nd	nd	Recovery
Nicholls et al. (104)	2	nd	High	IgG	anti-A, anti-A and anti-D	nd	nd	nd
Kim et al. (105)	2	B+	High	IgG	anti-B	nd	nd	nd
Brox et al. (106)	1	nd	High	IgG	anti-A	nd	nd	nd

*Clinical and immunological characteristics of patients described in case reports. Numbers in parenthesis indicate the number of patients with the given condition*.

A significant proportion of patients receiving IVIG develop a positive direct antiglobulin test (DAT) detectable after 24 h for up to 10 days after the IVIG infusion (109, 110). However, it should be underlined that the DAT positivity due to the factors mentioned above (111, 112) is not sufficient *per se* to diagnose hemolysis and DAT positivity does not necessarily imply the presence of active hemolysis. DAT-positive mild hemolytic reactions can be easily missed and the true incidence of such reactions is difficult to document without careful clinical and laboratory follow-up.

In the majority of reports on HA, intravascular red blood cell (RBC) destruction via complement activation or extravascular RBC sequestration and removal by the reticulo-endothelial system was proposed to result from IgG alloantibodies with specificity for RBC antigens A, B, D, or C.

**Figure 7:**
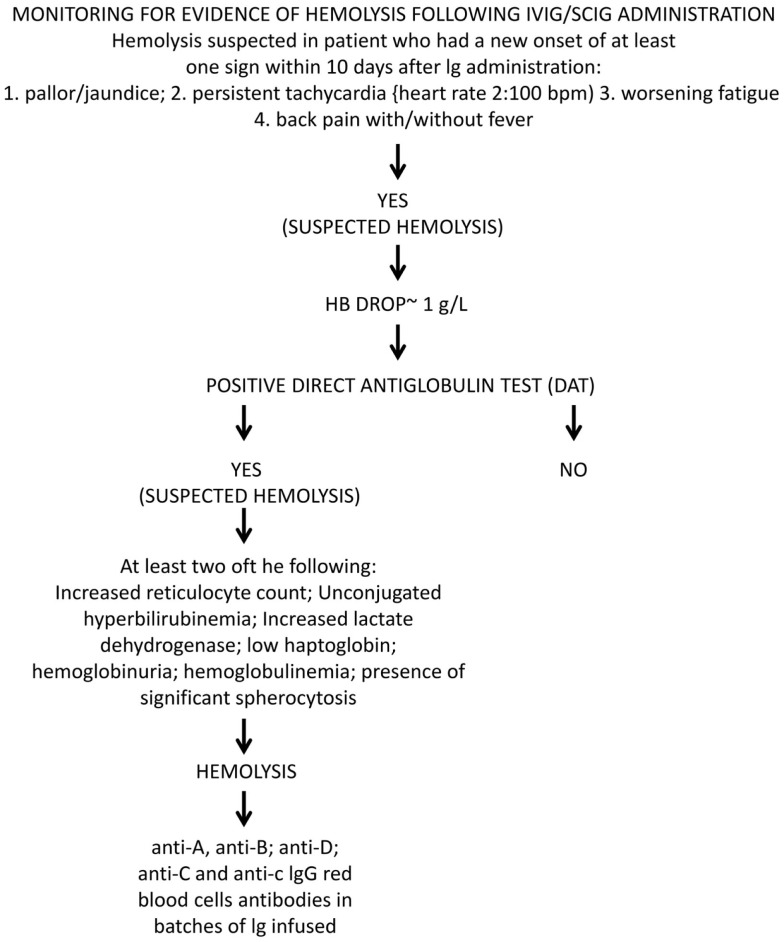
**Monitoring for evidence of oxygen missing at the dark side**. Diagnostic algorithm for Ig-induced hemolysis according to criteria elaborated by the Canadian Group (83).

Hemolytic anemia induced by high-dose IVIG has an average incidence of 5.8% (85). Low-dose IgG replacement therapy is considered universally as safe, and only few cases of hemolysis following low-dose IVIG or SCIG administration have been described (80, 95, 110). A baseline WBC and RBC count prior to IVIG initiation and a close clinical and laboratory follow-up was suggested as a useful tool for early diagnosis and treatment. A possible work up might be to check hemoglobin (Hb) level prior and 48–78 h after Ig infusion. In case of a drop of Hb, the presence of DAT, an increase in unconjugated bilirubin, lactate dehydrogenase (LDH), and reduced haptoglobin level, followed by a rise in reticulocyte count should be assessed (Figure 7). We systematically reviewed case reports related to IVIG-induced hemolysis from 1987 to 2014 and identified 29 articles containing reports of 109 patients. Baseline characteristics of the patients are shown in Table 2. When available, blood group, DAT, Hb drop, and outcome are indicated. All reports showed positive DAT, except for a case of Yin et al. (87); in this case, DAT was performed 10 days after IVIG administration and the DAT negativity might have been due to a rapid removal of sensitized RBCs.

In the majority of patients, the outcome was positive: 106 out of 109 patients recovered with or without packed RBC transfusions; three patients died after HA, with the hemolytic episode representing a precipitating factor of a severe underlying condition. Elution experiments were performed and the search for blood group antibodies revealed anti-A and anti-B specificity in the majority of cases; anti-D specificity was assessed in four reports, often associated with other specificities (95, 106, 110). A search for other specificities such as anti-band 3 or anti-Gal was not performed. Only one report detected anti-C specificity in three patients; in one of them associated with anti-D irregular antibodies (110). Although studies were restricted to blood group antibodies, this finding demonstrated that polyvalent IgG preparations might contain clinically significant non-blood group antibodies, which are not part of the lot-release criteria in that their titration is not yet required by the European Pharmacopeia. Antibodies in HA, such as anti-C, may have unexpected hemolytic consequences (113–117). Beside passive transfer of alloantibodies, IgG administration also has been demonstrated to lead to unspecific enhanced erythrocyte sequestration, in particular, in patients with underlying inflammatory disorders (109, 118). In 2009, the Canadian IVIG Hemolysis Pharmacovigilance Group elaborated criteria to define an “IVIG-induced hemolysis” (83). They included a reduction of Hb levels ≥1 g within 10 days after Ig administration, with appearance of a positive DAT and, at least, two of the following criteria: increase in the reticulocyte count, elevation of LDH and unconjugated bilirubin serum levels, low haptoglobin, hemoglobinuria, hemoglobinemia, presence of significant spherocytosis, in the absence of alternative causes of anemia. The passive transfer of IgG alloantibodies through IgG concentrates is difficult to explain as polyvalent IgG is prepared from plasma of thousands of donors. Since immunization to RBC alloantigens can occur because of past transfusions or pregnancy, the hypothetical numbers of alloimmunized plasma donors should be rather low. Recently, other mechanisms underlying alloimmunization related to molecular mimicry have been demonstrated (119). The mechanism of high-dose IVIG-induced HA is complex and it might vary from patient to patient. IVIG cause hemolysis due to: (i) disease-associated pre-coating of RBCs; (ii) IgG with hemolysis triggered by passive transfer of IgG binding to blood group antigens; (iii) transfer of high levels of alloantibodies to RBC pre-coated at a low level only; or (iv) transfer of clinically tolerable levels of isoagglutinins plus transfer of additional RBC-reacting physiological autoantibodies. Indeed, hemolytic reactions could not be related exclusively to transfer of alloantibodies. Hence, antibodies other than histo-blood group alloantibodies (pre-)coated to RBCs might contribute to hemolysis in IgG recipients need to be identified. In addition, hemolytic episodes may possibly be precipitated by some sort of complexed/denatured IgG that co-purify with other IgG in the product (76, 109, 118, 120). Recently, a two hit mechanism for IVIG-induced hemolysis has been proposed: the passive transfer of alloantibodies through IVIG representing the first hit and the underlying inflammatory state representing the second hit (121). Nowadays all commercial Ig products have to undergo anti-A and anti-B testing and regulatory requirement ask for respective IgG antibody titers of ≤1:64 at 5% solution strength (w/v) (103, 104). Nevertheless, hemolysis might occur even in recipients of IgG products that meet these specifications (76). Consequently, it has been suggested that IgG recipients should be monitored for clinical signs and symptoms of hemolysis (122).

### Reduction of histo-blood group A and B alloantibodies in IgG concentrates raises the chance for staying on the sunny side of the moon

With the detection of the immunomodulatory potential of IgG concentrates, their clinical use has continuously increased (123). To cover the need, at a first glance, an increase of the volume of plasma fractionated seems to be the most convenient option. However, this might economically not be viable because fractionation of plasma products is interconnected (124) and before increasing output of one product (e.g., IVIG), the market absorbance of the other products as well (e.g., albumin) must be ascertained. On a longer-run, a more viable option is to improve recovery. Considering recovery, the cold-ethanol fractionation apparently has reached its limits. As of today, four manufactures have invested into a “modern” fractioning technique on the basis of ion-exchange chromatography. Ion-exchange chromatography allows elevated recovery at high purity. As of today, five IVIGs, one SCIG, and one anti-D concentrate are fractionated by ion-exchange chromatography. Pharmacovigilance has shown that all chromatographically fractionated IVIG and SCIG, more or less prominently, show a tendency for elevated frequencies of hemolytic AEs. Anti-A and anti-B alloantibody titers are now lot-release criteria (see above) as they constitute the major risk parameter for hemolytic reactions mediated by IgG concentrates. To overcome the threat of end up on the dark side of the moon, two manufacturers have taken measures to reduce anti-A and anti-B titers in their IgG products. One measure chosen was adsorption of the alloantibodies by affinity chromatography (125). Reported reduction in both alloantibodies was significant and levels were similar to those in cold-ethanol fractionated immunoglobulins (126). The other measure chosen was reduction in anti-A using an automated indirect agglutination test for donor screening and exclusion of high-titer donations (approximately 5.1%) from plasma pooling and fractionation (127). This measure reduced anti-A in the IgG concentrate by one titer step. To ensure staying on the safe and sunny side, the manufacturer has announced the introduction of an alloanti-A and alloanti-B immune-affinity chromatography step into the manufacturing process (128). Preliminary results indicate depletion in anti-A and anti-B by >80% in investigational lots. Subsequently, we want to discuss possible consequences of (extensive) removal of antibodies reacting with histo-blood group antigens A and B.

### Reasoning about antibodies reacting with terminal sugars of the major histo-blood group antigens A and B

Three facts have initiated our thinking about possible consequences of removal of histo-blood group A and B reacting antibodies from IgG concentrates. (I) In collaboration with Hans U. Lutz, formerly Biochemistry ETH Zurich, we have observed the non-intended removal of natural anti-C3 autoantibodies regulating complement activation by large-scale immune-affinity adsorption of IgA from an IgG concentrate (129). Anti-C3 antibodies belong to the family of “NAbs” and have a particular role in homeostasis: they control activation of complement, among others, in the frame of NAb-mediated opsono-phagocytosis of altered or senescent cells, including RBCs (130–132). Thus, the intention to target one particular antibody by affinity chromatography might reduce that antibody specificity but at the same time affect other specificities as well. (II) It should be kept in mind that the blood groups A and B are in fact “histo-blood group” antigens, i.e., they are also found on white blood cells, T lymphocytes, and proteins and also can be found in soluble form (133). Alloantibodies reacting with histo-blood group antigens A and B thus have much broader tissue recognition than RBCs only. In addition, alloanti-A and alloanti-B belong to the population of NAbs recognizing non-self and most likely participate in primary host defense (134). (III) In contrast to cold-ethanol fractionation, where low titers of alloanti-A and alloanti-B are achieved on basis of their isoelectric points (IEPs), the (extensive) immune-affinity removal might affect a much wider IEP range, thereby removing broadly reacting antibodies and impairing some desirable functions of the IgG concentrate. Thus, the struggle for staying on the sunny side of the moon might have consequences for the antibody repertoire in an IgG concentrate.

Antibodies reacting with terminal di-, tri-, and tetra-saccharides belong to the large family of human anti-glycan NAbs. Histo-blood group A and B epitopes in terminal position are tetra-saccharides. Alloantibodies to these tetra-saccharides are found in the plasma of healthy individuals depending on the blood group they have. A considerable body of research into the nature of these NAbs has been performed so far, all using for isolation the corresponding terminal di- or tri-saccharides (135–137). Recently, the repertoire and epitope specificity of such immunoglobulins was addressed in depth by including the tetra-saccharide as well (138, 139). It proved that serum of healthy individuals contain respectable amounts of di- or tri-saccharide-reacting NAbs. These NAbs proved to be pseudo-anti-A and pseudo-anti-B NAbs as they are not reacting with the tetra-saccharide of histo-blood groups A and B. In contrast, alloanti-A and -B antibodies able to react with tetra-saccharides are reacting with the corresponding terminal di- and tri-saccharides. Reasoning about the biological role of these “high-titer and population conservative” anti-di- and anti-tri-saccharide NAbs and the consequence of their potential removal by immunoaffinity is outlined below.

A population of the anti-glycan NAbs are the anti-αGal NAbs which recognize Galα1-3Gal and Galα1-3(Fucα1-2)Gal epitopes. Anti-αGal NAbs have been described being xenoreactive, recognizing bacterial Galα1-3Gal (140) and having tissue homeostatic function. The daily removal of altered/senescent cells of the body is ~10^12^. Removal is mainly mediated by apoptosis (no inflammation, no necrosis). RBCs, when they do not encounter a pathological condition, over their life span of 100–120 days remain intact although they shrink, do not undergo apoptosis but progressively become senescent, mainly due to cumulative oxidative stress. Removal of intact RBCs with a daily turnover of ~2 × 10^11^, corresponding to ~20 g cell mass, is effectuated by increased exposure of otherwise cryptic structures such as spectrin, band 3, or αGal epitopes. These exposed structures are recognized by low affinity, high avidity, C3-bearing NAbs, which promote the efficient removal of intact senescent RBCs (130, 141, 142). Immunoaffinity adsorption by tri-saccharides columns of di- and tri-saccharide reacting NAbs from IgG concentrates can eliminate anti-histo-blood A and B alloantibodies while it also eliminates αGal and this might have a Janus effect. The face directed to the sun tells that adsorbing αGal NAbs reacting with altered and senescent self on RBC might prevent an increase in the IgG load of RBCs over the threshold level of relevant hemolysis in individuals at risk. The face directed to the dark indicate that adsorption of tissue homeostatic antibodies might deprive an IgG concentrate of potentially beneficial antibodies. Although they are NAbs, tri-saccharide reacting antibodies can be induced by feeding bacteria bearing the corresponding carbohydrate epitopes (134). These inducible NAbs are considered to participate in primary host defense. Other antibodies possibly involved in primary host defense are the anti-αGal NAbs. They show a broad specificity and can react with a number of related αGal-terminated oligosaccharides, including those on bacteria (143). Thus, the immunoadsorption of di-and tri-saccharide reacting NAbs might diminish the potential of an IgG concentrate to mediate primary host defense. Therefore, when choosing affinity resins for immunoadsorption, there might be some aspects worth to consider.

In summary, the principles of avoiding co-fractionation through cold-ethanol fractionation (144) versus immune-affinity removal of histo-blood group alloantibodies can have an impact on the presence of homeostatic and first-line defense antibodies. According to present knowledge, only resins coated with the corresponding tetra-saccharides can ascertain the selective removal of histo-blood group alloantibodies presumably involved in HA. Resins coated with the corresponding di- and tri-saccharides also remove blood group alloantibodies, however not selectively. Such resins in addition might remove a broad range of NAbs present in IgG concentrates at relative high amounts. In the literature, the use of tri-saccharide-coated resin was reported (145–147). We have found no information available in the public domain indicating which type of resin is/will be used for reduction of the histo-blood group alloantibodies in large-scale fractionation of IgG. Furthermore, we suggest that the effect of reduction of anti-A and ant-B reacting antibodies by immune-affinity on the antibody repertoire of IgG concentrates can only be assessed by, e.g., using pathogens/commensals, which share the saccharide epitopes, that have been used to coat the affinity resins or alternatively by exposing senescent RBCs stripped off the IgGs coated *in vivo*. Finally, techniques are required, which allow detection of low affinity, high avidity NAbs.

## Thrombosis – Falling into a Dark Lunar Crater

IVIG administration-related AEs, including thrombosis, have been extensively described (148). Thrombotic AEs are severe AEs and patients with risk factors require a special care. Reported average incidence of IVIG-induced thrombosis ranges from 3 to 13% (149). Recognized risk factors for IVIG-induced thrombosis include male gender; age >60; diabetes; renal insufficiency, dyslipidemia; hypertension; immobility; coronary disease; pre-existing vascular disease, family history of early thromboembolic disease; atrial fibrillation, high-dose and high-speed IVIG infusions. IVIG-induced thrombosis is reported both as venous events such as thrombosis stroke, pulmonary embolism (PE), deep venous thrombosis (DVT), and arterial ischemia events such as myocardial infarct and stroke. The mechanisms leading to IVIG-associated thrombosis are still not completely clear; three main mechanisms have been proposed, emphasizing the role of an increased blood viscosity causing a hypercoagulable state (150), the role of anticardiolipin antibodies passively transferred through IVIG (151), and the role of factor XIa or other biologically highly active factors passively transferred via IgG concentrates, such as PKA. Avoiding activated coagulation factors in IgG concentrates starts with appropriate anticoagulation of donated blood/plasma, i.e., careful mixing of anticoagulant and sample over the whole donation process. Alterations in an established manufacturing process neglecting appropriate controls can also lead to increased risk of transmission of activated coagulation factors. High MW proteins passively transferred by IVIG are probably contributing to this phenomenon (152). In patients with other risk factors, such as vascular disease, the increase in blood viscosity can precipitate thromboembolic events. As elderly individuals are prone for such AEs, we like to point to the possibility of elevated altered/senescent self-reacting with infused homeostatic NAbs being a possible factor facilitating thrombotic events as well. A relationship between IVIG administration and cerebral vasospasm has also been suggested by Sztajzel et al. (153); blood viscosity is a determinant for oxygen delivery to the tissues, and changes in viscosity can lead to a reduction in cerebral or myocardial perfusion.

**Figure 8:**
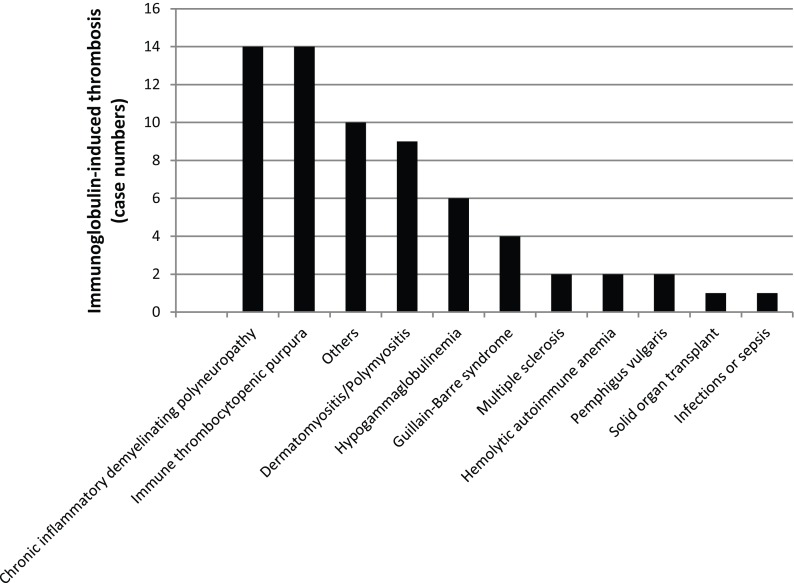
**Falling into a dark lunar crater while being on Ig treatment**. Bars indicate the number of patients with a given clinical condition and Ig-mediated thrombosis.

We systematically reviewed case reports related to IVIG-induced thrombosis from 1986 to 2014 (Figure 8). Literature search identified 35 articles containing reports concerning 65 patients (6, 24, 149, 154–183). When data were available, diagnosis, risk factors, the number of IVIG infusion prior to thrombosis event, and outcome have been indicated. Baseline characteristics of the patients are shown in Table 3. High-dose IVIG induced thromboembolic events in 59 patients at low to medium IVIG doses. Marie et al. (163) observed that the frequency and type of arterial events was inversely related to the time elapsed from IVIG infusion; almost 50% (23 versus 21 reports) of arterial ischemic events occurred within 12 h following IVIG, while about 75% of venous thrombosis occurred after more than 24 h. No correlation between number of infusions and occurrence of AE was observed. The main risk factors observed in this review were hypertension (19 cases, 33% of prevalence), previous vascular disease (18%), and dyslipidemia (17%). Average mortality for thrombotic events was 10% (arterial ischemia 9% versus venous thrombosis 11%, PE representing the main venous fatal event). Predicting IVIG-induced thrombosis is difficult. Risk factors should be assessed for each patient including instrumental exams when needed. Doppler ultrasound can be useful as early diagnostic tool for thrombosis or to detect the presence of abnormal blood flow especially after prolonged immobility. IVIG should be administered at low IR to reduce the risk. The administration of antiplatelet or anticoagulant prophylaxis was suggested in patients with several risk factors (162). However, thrombotic events have been reported even after several previous uncomplicated courses of treatment. In such cases, patients should be examined for signs and symptoms of thrombosis during each courses of IVIG.

**Table 3:** **Falling into a deep lunar crater – Ig-induced thrombosis in recipients of polyvalent immunoglobulins**.

Publication	Number of patients	Age	Diagnosis	Ig dosage	Predisposing factors	Number of IVIG infusion prior to thrombosis event	Thrombosis (arterial or venous)	Time from the last infusion	Outcome
Vinod et al. (154)	1	>65	Guillain-Barrè	High		First	Arterial	72 h	Recovery
Sin et al. (157)	1	<65	Solid organ transplantation	High		First	Arterial	48 h	Recovery post-emergency renal transplant
Min et al. (24)	1	<65	CVID	Low (SCIG)	Hypercoagulability (oral contraceptive)	Several	Venous		nd
Rajabally et al. (149)	5	<65 (2), >65 (3)	CIDP	High	Diabetes (2), hypertension (2), immobility (4), coronary disease (2), arrhythmia (1)	First (3), several (2)	Arterial (3), venous (2)	14 days	Recovery (4), death (1)
Al-Riyami et al. (156)	1	11	ITP	High		Several	Venous	10 days	Recovery
Iroh et al. (155)	1	13	ITP	High	Estrogen treatment	First	Venous	12 h	Death
Barada et al. (158)	1	11	XLA	Low		Several	Venous	nd	nd
Lee et al. (159)	1	56	ITP	High		First	Venous	72 h	Recovery
White et al. (160)	1	43	Dermatomyositis	High		First	Arterial	2 h	Recovery
Feuillet et al. (161)	1	38	Multiple sclerosis	High	Oral contraceptives	Seventh	Venous	6 days	Recovery
Marie et al. (162)	2	51, 55	Polyarteritis nodosa (1), polymyositis (1)	High		Third, 15th	Venous (2)	2 h, 7 days	Recovery
Marie et al. (163)	6	76, 49, 63, 45, 64, 64	AHA (1), polymyositis (5)	High	Hypertension (3), hypercholesterolemia (3)	Second, sixth, several (4)	Venous (3), arterial (3)	2 days, 6 h (5)	Recovery
Geller et al. (165)	1	28	Streptococcal toxic shock syndrome	High		First	Venous	8 days	Recovery
Sheehan et al. (167)	1	43	*Pemphigus vulgaris*	High	Immobility, hypertension	First	Venous	16 days	Recovery
Hefer et al. (164)	1	85	ITP	High	Hypertension, chronic myelogenous leukemia	Second	Arterial	3 h	Recovery
Feuillet et al. (166)	1	38	Multiple sclerosis	High	Oral contraceptives	First	Venous	nd	Recovery
Vucic et al. (168)	7	57, 69, 75, 81, 79, 62, 80	CIPD (4), anti-MAG neuropathy (1), multifocal motor neuropathy (1)	High	Hypertension (3), hypercholesterolemia (3), previous stroke (2), arrhythmia (1)	Second (1), third (2), several (5)	Arterial (6), venous (1)	1 h (2), 2 days (3), 2 weeks (2)	Recovery (5)
Stamboulis et al. (169)	1	36	CIPD	High	Heavy smoker	First	Arterial	nd	Recovery
Katz et al. (170)	2	67, 65	*Pemphigus vulgaris*, dermatomyositis	High	Hypertension	Second, first	Arterial (1), venous (1)	6 h	Recovery
Zaidan et al. (171)	3	47, 65, 70	GBS (1), CIDP (2)	High	Hypercholesterolaemia (1), diabetes (2)	Third (1), several (2)	Arterial (3)	1 h (2), 1 day (1)	Recovery (2)
Brown et al. (172)	3	70, 91, 42	CVID	Low	Diabetes (1), myocardial infarction (2)		Arterial (3)	6 h	Recovery
Evangelou et al. (173)	1	54	CVID	Low	High platelets count	Several	Venous	24 h	nd
Emerson et al. (174)	2	54, 33	ITP, Evans Syndrome	High	Obesity (1)	Second	Arterial (2); venous (1)	2 h, 2 days	Death (1), recovery (1)
Alliot et al. (175)	1	63	ITP	High	Hypertension	Fifth	Venous	3 days	Death
Sherer et al. (176)	2			High			Venous		
Elkayam et al. (177)	4	60, 41, 67, 67	ITP, polymyositis, connective disease, CIPD	High	Hypercholesterolemia (2), hypertension (2)	several (3), first (1)	Arterial	4 days	Recovery
Go et al. (178)	1	52	ITP	High		First	Venous	1 day	Recovery
Turner et al. (179)	1	60	Miller-Fisher syndrome	High		Fifth	Arterial	5 days	Recovery
Harkness et al. (180)	1	40	CIDP	High	Hypercholesterolemia		Arterial and venous	7 days	Recovery
Paolini et al. (181)	1	78	ITP	High	Hypercholesterolemia	First	Arterial	7 days	Recovery
Rosenbaum et al. (182)	1	76	Miller-Fisher syndrome	High		First	Arterial	12 h	Recovery
Oh et al. (183)	1	17		High		Several	Venous	1 day	Recovery
Dalakas et al. (150)	2	62, 52		High		Second	Venous (1), arterial (2)	7 days, 2 days	Death (1), recovery (1)
Woodruff et al. (6)	4	72, 73, 62, 83	ITP	High	Hypertension (3), obesity (3), previous stroke (2), previous myocardial infarction (1)	Several (4)	Arterial (4)	2 h (3), 2 days (1)	Death (3)

*Clinical and immunological characteristics of patients described in case reports*.

*Numbers in parenthesis indicate the number of patients with the given condition*.

Immunoglobulin G concentrates are widely acknowledged to offer a safe, high-dose, long-term therapy option for a variety of diseases. AEs occur rarely and mainly are mild to moderate. Deviations from this rule of thumb are addressed by authorities and the plasma fractionation industry to achieve corrections. Above, we have reviewed two types of AE which have shown elevated frequency in the near past. We tried to give some insights which might help in reducing frequencies of AEs bed side.

## Conflict of Interest Statement

The authors declare that the research was conducted in the absence of any commercial or financial relationships that could be construed as a potential conflict of interest.

## Abbreviations

ACA, anti-complementary activity = unwanted spontaneous complement-activating capacity of an Ig concentrate; AE, adverse event; BCR, B cell receptor; BT, body temperature; C3a/C3a[desArg], anaphylatoxin C3a and its arginine deprived form which were detected together; CH50, total complement hemolytic activity 50%; cGMP, current good manufacturing practice; CVID, common variable immunodeficiency; DAT, direct antiglobulin test; DVT, deep venous thrombosis; HA, hemolytic anemia; Hb, hemoglobin; HR, heart rate; IC, immune complex; IgA, immunoglobulin A; IgA1, subclass 1 of IgA; IgA2, subclass 2 of IgA; IgAD, IgA deficiency; IgG, immunoglobulin G; IgM, immunoglobulin M; IL-1ra, interleukin 1 receptor antagonist; IL-6, interleukin 6; IL-8, interleukin 8; IMIG, immunoglobulin G concentrate for intramuscular application; IR, infusion rate; IVIG, immunoglobulin G concentrate for intravenous application; IEP, isoelectric point; ITP, immune thrombocytopenic purpura; LDH, lactate dehydrogenase; MERS, middle east respiratory syndrome; MW, molecular weight; NAb, natural antibody or autoantibody; PKA, prekallikrein activator; PE, pulmonary embolism; RBC, red blood cell, erythrocyte; SARS, severe acute respiratory syndrome; SCIG, immunoglobulin concentrate for subcutaneous application; sIgAD, selective IgAD; TCR, T cell receptor; TNFα, tumor necrosis factor alpha; V-region, variable region of antibodies, TCR and BCR; vCJD, variant Creutzfeldt-Jakob disease.

## References

[1] John TJ Ninan GT John F Flewett TH Francis DP Zuckerman DP. Epidemic hepatitis B caused by commercial human immunoglobulin. Lancet (1979) 1:1074. 10.1016/S0140-6736(79)92964-7 86788

[2] Barandun S Kistler P Jeunet F Isliker H Intravenous administration of human γ-globulin. Vox Sang (1962) 7:157–74 10.1111/j.1423-0410.1962.tb03240.x 13864762

[3] Ellis EF Henney CS Adverse reactions following administration of human gamma-globulin. J Allergy (1969) 43:45–54 10.1016/0021-8707(69)90019-7 4178567

[4] Kamme C Dahlquist E Jonsson S Lindström F IgG antibodies to IgA in two patients with hypogammaglobulinemia treated with commercial gammaglobulin. Acta Pathol Microbiol Immunol Scand (1975) 83:189–94.

[5] Alving BM Tankersley DL Mason BL Rossi F Aronson DL Finlayson JS. Contact-activated factors: contaminants of immunoglobulin preparations with coagulant and vasoactive properties. J Lab Clin Med (1980) 96:334–46. 6447181

[6] Woodruff RK Grigg AP Firkin FC Smith IL Fatal thrombotic events during treatment of autoimmune thrombocytopenia with intravenous immunoglobulin in elderly patients. Lancet (1986) 2:217–8 10.1016/S0140-6736(86)92511-0 2873457

[7] Reinhart WH Berchtold PE. Effect of high-dose intravenous immunoglobulin therapy on blood rheology. Lancet (1992) 339:662–4. 10.1016/0140-6736(92)90806-E 1347348

[8] Späth PJ Van Holten RW Kempf C Pathogen safety of immunoglobulin preparations. 2nd ed In: Wahn V Orange J, editors. Clinical Use of Immunoglobulins. Bremen: UNI-MED Verlag (2013). p. 26–50.

[9] Jackson GS Burk-Rafel J Edgeworth JA Sicilia A Abdilahi S Korteweg J Population screening for variant Creutzfeldt-Jakob disease using a novel blood test: diagnostic accuracy and feasibility study. JAMA Neurol (2014) 71:421–8. 10.1001/jamaneurol.2013.6001 24590363 PMC4158718

[10] Kaveri SV. Intravenous immunoglobulin: exploiting the potential of natural antibodies. Autoimmun Rev (2012) 11:792–4. 10.1016/j.autrev.2012.02.006 22349620

[11] Baumgarth N Tung JW Herzenberg LA. Inherent specificities in natural antibodies: a key to immune defense against pathogen invasion. Springer Semin Immunopathol (2005) 26:347–62. 10.1007/s00281-004-0182-2 15633017

[12] Terness P Kohl I Hubener G Battistutta R Moroder L Welschof M The natural human IgG anti-F(ab’)2 antibody recognizes a conformational IgG1 hinge epitope. J Immunol (1995) 154:6446–52. 7539020

[13] Lutz HU Fumia S. Stimulation of complement amplification by F(ab’)_2_-containing immune complexes and naturally occurring anti-hinge antibodies – possible role in systemic inflammation. Autoimmunity (2008) 7:508–13. 10.1016/j.autrev.2008.04.017 18558371

[14] Bouhlal H Martinvalet D Teillaud J-L Fridman C Kazatchkine MD Bayry J Natural autoantibodies to Fcγ receptors in intravenous immunoglobulins. J Clin Immunol (2014) 34(Suppl 1):S4–11. 10.1007/s10875-014-0019-2 24682714

[15] Bouhlal H Kaveri S. Multi-faceted role of naturally occurring autoantibodies in fighting pathogens. Adv Exp Med Biol (2012) 750:100–13. 10.1007/978-1-4614-3461-0_8 22903669

[16] Kazatchkine MD Kaveri SV Immunomodulation of autoimmune and inflammatory diseases with intravenous immune globulin. N Engl J Med (2001) 345:747–55 10.1056/NEJMra993360 11547745

[17] Negi VS Elluru S Sibéril S Graff-Dubois S Mouthon L Kazatchkine MD Intravenous immunoglobulin: an update on the clinical use and mechanisms of action. J Clin Immunol (2007) 27:233–45. 10.1007/s10875-007-9088-9 17351760

[18] Harrington WJ Minnich V Hollingsworth JW Moore CV Demonstration of a thrombocytopenic factor in the blood of patients with thrombocytopenic purpura. J Lab Clin Med (1951) 38:1–10. 14850832

[19] Imbach P Barandun S D’Apuzzo V Baumgartner C Hirt A Morell A High-dose intravenous gammaglobulin for idiopathic thrombocytopenic purpura in childhood. Lancet (1981) 1:1228–31. 10.1016/S0140-6736(81)92400-4 6112565

[20] Altznauer F von Gunten S Späth P Simon HU. Concurrent presence of agonistic and antagonistic anti-CD95 autoantibodies in intravenous Ig preparations. J Allergy Clin Immunol (2003) 112:1185–90. 10.1016/j.jaci.2003.09.045 14657880

[21] Casulli S Topcu S Fattoum L von Gunten S Simon HU Teillaud JL A differential concentration-dependent effect of IVIg on neutrophil functions: Relevance for anti-microbial and anti-inflammatory mechanisms. PLoS ONE (2011) 6:e26469 10.1371/journal.pone.0026469 22065996 PMC3204983

[22] Späth P Kempf C Gold R Herstellung, Verträglichkeit und Virussicherheit von intravenösen Immunglobulinen. 1st ed In: Berlit P, editor. Immunglobuline in der klinischen Neurologie. Darmstadt: Steinkopff (2001). p. 1–42.

[23] Divan HA Menis M Sridhar G Selvam N Forshee R Izurieta HS Occurrence of hemolytic reactions (HRS) on the same day as immune globulin (IG) product administrations during 2008–2012 [abstract]. Pharmacoepidemiol Drug Saf (2013) 25:436 10.1002/pds.3512

[24] Min J Bhatt A Aburashed R Burton S. Cerebral venous and sinus thrombosis associated with subcutaneous immunoglobulin injection and oral contraceptive use. Neurol Sci (2012) 33:627–9. 10.1007/s10072-011-0778-y 21915646

[25] Moltenis M Valnet Rabier M Rohrlich P Kantelip J Multiple immunoglobulin intolerance without antibody’s anti-immunoglobulin A: a case report [abstract]. Fundam Clin Pharmacol (2011) 25(Suppl 1):72 10.1111/j.1472-8206.2011.00930.x 20070855

[26] Thobani S Hu E Huynh P Scott L Common variable immunodeficiency: a patient with anaphylaxis to intravenous and subcutaneous immunoglobulin. J Allergy Clin Immunol (2010) 125:AB143 10.1016/j.jaci.2009.12.559

[27] Quinti I Soresina A Agostini C Spadaro G Matucci A Sfika I Prospective study on CVID patients with adverse reactions to intravenous or subcutaneous IgG administration. J Clin Immunol (2008) 28:263–7. 10.1007/s10875-007-9169-9 18214651

[28] Richard C Michael B Severe adverse reaction to subcutaneous immunoglobulin therapy in a patient with common variable immunodeficiency [abstract]. Clin Exp Immunol (2013) 174(Suppl 1):33 10.1111/cei.12218

[29] Zenker O Rojavin M Baggish J Bexon M Evaluation of the relationship between injection site reaction rate and SCIg doses in patients with primary immunodeficiencies. J Allergy Clin Immunol (2012) 129(2 Suppl1):AB194 10.1016/j.jaci.2011.12.287

[30] Barandun S Morell A Adverse reactions to immunoglobulin preparations. In: Nydegger UE, editor. Immunthemotherapy: A Guide to Immunoglobulin Prophylaxis and Therapy. London: Academic Press (1981). p. 223–7.

[31] Björkander J Wadsworth C Hanson LA. 1040 Prophylactic infusions with an unmodified intravenous immunoglobullin product causing few side-effects in patients with antibody deficiency syndromes. Infect (1985) 13:102–10. 10.1007/BF01642867 4030106

[32] Rachid R Bonilla FA. The role of anti-IgA antibodies in causing adverse reactions to gamma globulin infusion in immunodeficient patients: a comprehensive review of the literature. J Allergy Clin Immunol (2012) 129:628–34. 10.1016/j.jaci.2011.06.047 21835445

[33] Lu P Ling B Li R Prevalence of immunoglobulin a deficiency (IgAD) in Shanghai blood donors and efforts to establish a rare blood bank of IgAD in Shanghai [abstract]. Transfusion (2013) 53:98A–9A 10.1111/trf.12401

[34] IUIS Scientific Committee. Primary immunodeficiency diseases. Report of an IUIS Scientific Committee. International Union of Immunological Societies. Clin Exp Immunol (1999) 118(Suppl 1):1–28 10.1046/j.1365-2249.1999.00109.x PMC1905383 10540200

[35] Hammarström L Persson MAA Smith CIE. Anti-IgA in selective IgA deficiency – in vitro effects and Ig subclass pattern of human anti-IgA. Scand J Immunol (1983) 18:509–13. 6607511 10.1111/j.1365-3083.1983.tb00885.x

[36] Björkander J Hammarström L Smith CI Buckley RH Cunningham-Rundles C Hanson LA. Immunoglobulin prophylaxis in patients with antibody deficiency syndromes and anti-IgA antibodies. J Clin Immunol (1987) 7:8–15. 10.1007/BF00915419 3494039

[37] Ferreira A Garcia Rodriguez MC Fontán G. Follow-up of anti-IgA antibodies in primary immunodeficient patients treated with gamma-globulin. Vox Sang (1989) 56:218–22. 10.1111/j.1423-0410.1989.tb02032.x 2474899

[38] Burks AW Sampson HA Buckley RH Anaphylactic reactions after gamma globulin administration in patients with hypogammaglobulinemia: detection of IgE antibodies to IgA. N Engl J Med (1986) 314:560–4 10.1056/NEJM198602273140907 3945295

[39] Tinegate HN Ball J Poles D Regan F Sewell C Bolton-Maggs P Management of immunoglobulin A deficiency: lessons from haemovigilance [abstract]. Vox Sang (2013) 105(S1):23 10.1111/vox.12047

[40] Palmer DS O’Toole J Montreuil T Scalia V Yi QL Goldman M Screening of Canadian blood services donors for severe immunoglobulin A deficiency. Transfusion (2010) 50:1524–31. 10.1111/j.1537-2995.2010.02588.x 20158683

[41] Ropars C Müller A Paint N Beige D Avenard G. Large scale detection of IgA deficient blood donors. J Immunol Methods (1982) 54:183–9. 10.1016/0022-1759(82)90059-X 6983542

[42] Strobel E von Meyer A. Unexpected reactions of the anti-IgA antibody particle gel immunoassay. Transfus Med (2014) 24:55–7. 10.1111/tme.12094 24325384

[43] Robitaille N Delage G Long A Thibault L Robillard P. Allergic transfusion reactions from blood components donated by IgA-deficient donors with and without anti-IgA: a comparative retrospective study. Vox Sang (2010) 99:136–41. 10.1111/j.1423-0410.2010.01326.x 20345516

[44] Horn J Thon V Bartonkova D Salzer U Warnatz K Schlesier M Anti-IgA antibodies in common variable immunodeficiency (CVID): diagnostic workup and therapeutic strategy. Clin Immunol (2007) 122:156–62. 10.1016/j.clim.2006.10.002 17137841

[45] Sundin U Nava S Hammarström L. Induction of unresponsiveness against IgA in IgA-deficient patients on subcutaneous immunoglobulin infusion therapy. Clin Exp Immunol (1998) 112:341–6. 10.1046/j.1365-2249.1998.00571.x 9649200 PMC1904967

[46] van der Heijden J Geissler J van Mirre E van Deuren M van der Meer JWM Salama A A novel splice variant of FcgRIIa: a risk factor for anaphylaxis in patients with hypogammaglobulinemia. J Allergy Clin Immunol (2013) 131:1408.e–16.e. 10.1016/j.jaci.2013.02.009 23545275

[47] Aukrust P Frøland SS Liabakk NB Müller F Nordoy I Haug C Release of cytokines, soluble cytokine receptors, and interleukin-1 receptor antagonist after intravenous immunoglobulin administration *in vivo*. Blood (1994) 84:2136–43. 7919327

[48] Berkovitch M Dolinski G Tauber T Aladjem M Kaplinsky C. Neutropenia as a complication of intravenous immunoglobulin (IVIG) therapy in children with immune thrombocytopenic purpura: common and non-alarming. Int J Immunopharmacol (1999) 21:411–5. 10.1016/S0192-0561(99)00020-X 10405875

[49] Bolli R Brügger R Hodler G Maeder W Spycher MO Gennari K IgG dimers in liquid intravenous immunoglobulin preparations. In: Kazatchkine MD Morell A, editors. Intravenous Immunoglobulin – Research and Therapy. London: The Parthenon Publishing Group (1996). p. 307–8.

[50] Schnorf J Arnet B Burek-Kozlowska A Gennari K Rohner R Späth PJ Laboratory parameters measured during infusion of immunoglobulin preparations for intravenous use and related tolerability. In: Kazatchkine MD Morell A, editors. Intravenous Immunoglobulin – Research and Therapy. London: The Parthenon Publishing Group (1996). p. 312–3.

[51] Spycher MO Bolli R Hodler G Gennari K Hubsch A Späth P Well-tolerated liquid intravenous immunoglobulin G preparations (IVGG) have a low immunoglobulin G dimer (IgG-dimer) content. J Autoimmun (1999) 96(Suppl 1):S96A.

[52] Bleeker WK Agterberg J Rigter G de Vries van Rosen A Bakker JC. An animal model for the detection of hypotensive side effects of immunoglobulin preparations. Vox Sang (1987) 52:281–90. 10.1111/j.1423-0410.1987.tb04894.x 3498259

[53] Bleeker WK Agterberg J Rigter G Van Rooijen N Bakker JC. Key role of macrophages in hypotensive side effects of immunoglobulin preparations. Studies in an animal model. Clin Exp Immunol (1989) 77:338–44. 2805404 PMC1542048

[54] Bleeker WK Teeling JL Verhoeven AJ Rigter GM Agterberg J Tool AT Vasoactive side effects of intravenous immunoglobulin preparations in a rat model and their treatment with recombinant platelet-activating factor acetylhydrolase. Blood (2000) 95:1856–61. 10688848

[55] Teeling JL Bleeker WK Rigter GM Van Rooijen N Kuijpers TW Hack CE. Intravenous immunoglobulin preparations induce mild activation of neutrophils in vivo via triggering of macrophages – studies in a rat model. Br J Haematol (2001) 112:1031–40. 10.1046/j.1365-2141.2001.02674.x 11298603

[56] Kroez M Kanzy EJ Gronski P Dickneite G. Hypotension with intravenous immunoglobulin therapy: importance of pH and dimer formation. Biologicals (2003) 31:277–86. 10.1016/j.biologicals.2003.09.001 14624798

[57] Teeling JL Jansen-Hendriks T Kuijpers TW De Haas M van de Winkel JG Hack CE Therapeutic efficacy of intravenous immunoglobulin preparations depends on the immunoglobulin G dimers: studies in experimental immune thrombocytopenia. Blood (2001) 98:1095–9. 10.1182/blood.V98.4.1095 11493456

[58] Farber CM Crusiaux A Schandene L Van Vooren JP Goldman M Dupont E Tumor necrosis factor and intravenous gammaglobulins in common variable immunodeficiency. Clin Immunol Immunopathol (1994) 72:233–6. 10.1006/clin.1994.1136 8050197

[59] Bagdasarian A Tonetta S Harel W Mamidi R Uemura Y. IVIG adverse reactions: potential role of cytokines and vasoactive substances. Vox Sang (1998) 74:74–82. 10.1046/j.1423-0410.1998.7420074.x 9501404

[60] Michelis FV Branch DR Scovell I Bloch E Pendergrast J Lipton JH Acute hemolysis after intravenous immunoglobulin amid host factors of ABO-mismatched bone marrow transplantation, inflammation, and activated mononuclear phagocytes. Transfusion (2014) 54:681–90. 10.1111/trf.12329 23829192

[61] Ling ZD Yeoh E Webb BT Farrell K Doucette J Matheson DS Intravenous immunoglobulin induces interferon-γ and interleukin-6 *in vivo*. J Clin Immunol (1993) 13:302–9 10.1007/BF00920238 8245176

[62] Yoo EM Wims LA Chan LA Morrison SL. Human IgG2 can form covalent dimers. J Immunol (2003) 170:3134–8. 10.4049/jimmunol.170.6.3134 12626570

[63] Vassilev TL Bineva IL Dietrich G Kaveri SV Kazatchkine MD. Variable region-connected, dimeric fraction of intravenous immunoglobulin enriched in natural autoantibodies. J Autoimmun (1995) 8:405–13. 10.1006/jaut.1995.0032 7576001

[64] Simon HU Späth PJ IVIG – mechanisms of action. Allergy (2003) 58:543–52 10.1034/j.1398-9995.2003.00239.x 12823109

[65] Gronski P. IgG dimers in multidonor-derived immunoglobulins: aspects of generation and function. Curr Pharm Des (2006) 12:181–90. 10.2174/138161206775193154 16454735

[66] Tankersley DL Preston MS Finlayson JS. Immunoglobulin G dimer: an idiotype-anti-idiotype complex. Mol Immunol (1988) 25:41–8. 10.1016/0161-5890(88)90088-0 3343971

[67] Roux KH Tankersley DL. A view of the human idiotypic repertoire – electron microscopic and immunologic analyses of spontaneous idiotype-anti-idiotype dimers in pooled human IgG. J Immunol (1990) 144:1387–95. 2303712

[68] Gronski P Schridde C Forsterling HD. Polyreactive antibodies in multidonor-derived immunoglobulin G: theory and conclusions drawn from experiments. Immunobiology (2010) 215:356–69. 10.1016/j.imbio.2009.06.015 19592128

[69] Miescher SM Schaub A Ghielmetti M Baumann M Vogel M Bolli R Comparative analysis of antigen specificities in the monomeric and dimeric fractions of intravenous immunoglobulin. Ann N Y Acad Sci (2005) 1051:582–90 10.1196/annals.1361.102 16126998

[70] Wymann S Ghielmetti M Schaub A Baumann MJ Stadler BM Bolli R Monomerization of dimeric IgG of intravenous immunoglobulin (IVIg) increases the antibody reactivity against intracellular antigens. Mol Immunol (2008) 45:2621–8. 10.1016/j.molimm.2007.12.020 18280568

[71] Relkin NR Szabo P Rotondi M Mujalli D Antibodies in the dimer fraction of IVIg have the capacity to bind beta amyloid. Alzheimers Dement (2009) 5:427–8 10.1016/j.jalz.2009.04.95919751922

[72] Schaub A von Gunten S Vogel M Wymann S Stadler B Spycher M An analysis of anti-Fas and anti-Siglec-9 autoantibodies in monomeric and dimeric fractions of IVIG. Allergy (2009) 64:268–9. 10.1111/j.1398-9995.2011.02579.x 21385183

[73] Schaub A von Gunten S Vogel M Wymann S Rüegsegger M Stadler BM Dimeric IVIG contains natural anti-Siglec-9 autoantibodies and their anti-idiotypes. Allergy (2011) 66:1030–7. 10.1111/j.1398-9995.2011.02579.x 21385183

[74] Bridgham M Drake M Maguire K A case of haemolysis following administration of intravenous immunoglobulin [abstract]. Transfus Med (2014) 24:58 10.1111/tme.1213924283469

[75] Clemenz MR Joseph WM Shuler MJ Lynn AW. Intravenous immunoglobulin-induced hemolytic anemia in a patient with juvenile dermatomyositis. J Drugs Dermatol (2013) 12:111–3. 23377338

[76] Desborough MJ Miller J Thorpe SJ Murphy MF Misbah SA. Intravenous immunoglobulin-induced haemolysis: a case report and review of the literature. Transfus Med (2013) 24:219–26. 10.1111/tme.12083 24164446

[77] Mohamed M Bates G Eastley B Massive intravascular haemolysis after high dose intravenous immunoglobulin therapy. Br J Haematol (2013) 160:570 10.1111/bjh.12182 23294261

[78] Rink BD Gonik B Chmait RH O’Shaughnessy R. Maternal hemolysis after intravenous immunoglobulin treatment in fetal and neonatal alloimmune thrombocytopenia. Obstet Gynecol (2013) 121:471–3. 10.1097/AOG.0b013e3182765c63 23344412

[79] Berard R Whittemore B Scuccimarri R. Hemolytic anemia following intravenous immunoglobulin therapy in patients treated for Kawasaki disease: a report of 4 cases. Pediatr Rheumatol Online J (2012) 10:10. 10.1186/1546-0096-10-10 22507284 PMC3353234

[80] Pintova S Bhardwaj AS Aledort LM IVIG – a hemolytic culprit. N Engl J Med (2012) 367:974–6 10.1056/NEJMc1205644 22931280

[81] Morgan S Sorensen P Vercellotti G Zantek ND. Haemolysis after treatment with intravenous immunoglobulin due to anti-A. Transfus Med (2011) 21:267–70. 10.1111/j.1365-3148.2011.01078.x 21605202

[82] Welles CC Tambra S Lafayette RA. Hemoglobinuria and acute kidney injury requiring hemodialysis following intravenous immunoglobulin infusion. Am J Kidney Dis (2010) 55:148–51. 10.1053/j.ajkd.2009.06.013 19628320

[83] Canadian Blood Services. Important Information Regarding IVIG Associated Hemolysis (2014). Available from: http://www.bloodservices.ca/CentreApps/Internet/UW_V502_MainEngine.nsf/resources/CustomerLetters09/$file/CL_2009-02.pdf

[84] Gordon DJ Sloan SR de Jong JL A pediatric case series of acute hemolysis after administration of intravenous immunoglobulin. Am J Hematol (2009) 84:771–2 10.1002/ajh.21544 19806664

[85] Kahwaji J Barker E Pepkowitz S Klapper E Villicana R Peng A Acute hemolysis after high-dose intravenous immunoglobulin therapy in highly HLA sensitized patients. Clin J Am Soc Nephrol (2009) 4:1993–7. 10.2215/CJN.04540709 19833910 PMC2798878

[86] Daw Z Padmore R Neurath D Cober N Tokessy M Desjardins D Hemolytic transfusion reactions after administration of intravenous immune (gamma) globulin: a case series analysis. Transfusion (2008) 48:1598–601. 10.1111/j.1537-2995.2008.01721.x 18466176

[87] Yin F Nesbitt JA Tobian AA Holt PA Mikdashi J Hemolytic anemia following intravenous immunoglobulin administration. Am J Hematol (2008) 83:825 10.1002/ajh.21263 18756544

[88] Coghill J Comeau T Shea T Braddy L Bandarenko N Afenyi-Annan A Acute hemolysis in a patient with cytomegalovirus pneumonitis treated with intravenous immunoglobulin (IVIG). Biol Blood Marrow Transplant (2006) 12:786–8 10.1016/j.bbmt.2006.03.003 16785068

[89] Singh-Grewal D Kemp A Wong M. A prospective study of the immediate and delayed adverse events following intravenous immunoglobulin infusions. Arch Dis Child (2006) 91:651–4. 10.1136/adc.2005.078733 16638785 PMC2083046

[90] Chamouni P Tamion F Gueit I Girault C Lenain P Varin R Adverse effect of polyvalent immunoglobulin in the treatment of Guillain-Barré syndrome. Transfus Apher Sci (2003) 28:117–24. 10.1016/S1473-0502(03)00012-0 12679114

[91] Karaaslan S Oran B Calisman Ü Baysal T Baspinar O Tas A Hemolysis after administration of high-dose immunoglobulin in a patient with myocarditis. Turk J Haematol (2003) 20:237–40. 27263521

[92] Trifa M Simon L Hamza J Bavoux F des Roziers NB Haemolytic anaemia associated with high dose intravenous immunoglobulin therapy in a child with Guillain-Barré syndrome. Arch Dis Child (2003) 88:836–7 10.1136/adc.88.9.836-b 12937119 PMC1719657

[93] Ballot-Brossier C Mortelecque R Sinegre M Marceau A Dauriat G Courtois F Insisting on intravenous polyvalent immunoglobulin therapy in polymyositis in spite of the occurrence of sever hemolytic anemia – poursuite du traitement d’une polymyosite par les immunoglobulines intraveineuses polyvalentes malgré la survenue d’une anémie hémolytique sévère. Transfus Clin Biol (2001) 8:94–9. 11386046 10.1016/s1246-7820(01)00109-4

[94] Nagakawa M Watanabe N Okuno M Kondo M Okagawa H Taga T Severe hemolytic anemia following high-dose intravenous immunoglobulin administration in a patient with Kawasaki disease. Am J Hematol (2000) 63:160–1 10.1002/(SICI)1096-8652(200003)63:3<160::AID-AJH11>3.0.CO;2-4 10679809

[95] Wilson JR Bhoopalam H Fisher M. Hemolytic anemia associated with intravenous immunoglobulin. Muscle Nerve (1997) 20:1142–5. 10.1002/(SICI)1097-4598(199709)20:9<1142::AID-MUS8>3.0.CO;2-8 9270670

[96] Munoz J Garcia A Jordan C Rubio A Ferran C Martinez Matos JA Hemolysis during immunoglobulin therapy – hemolisis durante el tratamiento con inmunoglobulinas. Sangre (Barc) (1996) 41:72–3. 8779043

[97] Tamada K Kohga M Masuda H Hattori K. Hemolytic anemia following high-dose intravenous immunoglobulin administration. Acta Paediatr Jpn (1995) 37:391–3. 10.1111/j.1442-200X.1995.tb03339.x 7645396

[98] Boothe G Brecher ME Root M Robinson J Haley R Acute hemolysis due to passively transfused high-titer anti-B causing spontaneous in vitro agglutination. Immunohematology (1995) 11:43–5.

[99] Thomas MJ Misbah SA Chapel HM Jones M Elrington G Newsom-Davis J Hemolysis after high-dose intravenous Ig. Blood (1993) 82:3789. 8260715

[100] Comenzo RL Malachowski ME Meissner HC Fulton DR Berkman EM. Immune hemolysis, disseminated intravascular coagulation, and serum sickness after large doses of IVIG for Kawasaki disease. J Pediatr (1992) 120:926–8. 10.1016/S0022-3476(05)81964-X 1593353

[101] Rovelli A D’Angelo P Balduzzi A Borzini P Biondi A Uderzo C Acute intravascular haemolysis associated with high dose immunoglobulin after bone marrow transplantation for acute myelogenous leukemia. Leuk Lymphoma (1991) 5:71–4 10.3109/10428199109068107 27463212

[102] Okubo S Ishida T Yasunaga K. Hemolysis after intravenous immune globulin therapy: relation to IgG subclasses of red cell antibody. Transfusion (1990) 30:436–8. 10.1046/j.1537-2995.1990.30590296378.x 2360236

[103] Hillyer CD Schwenn MR Fulton DR Meissner HC Berkman EM. Autoimmune hemolytic anemia in Kawasaki disease: a case report. Transfusion (1990) 30:738–40. 10.1046/j.1537-2995.1990.30891020336.x 2219263

[104] Nicholls MD Cummins JC Davies VJ Greenwood JK. Haemolysis induced by intravenously-administered immunoglobulin. Med J Aust (1989) 150:404–6. 2469947 10.5694/j.1326-5377.1989.tb136537.x

[105] Kim HC Park CL Cowan JH III Fattori FD August CS. Massive intravascular hemolysis associated with intravenous immunoglobulin in bone marrow transplant recipients. Am J Pediatr Hematol Oncol (1988) 10:69–74. 10.1097/00043426-198821000-00012 3056063

[106] Brox AG Cournoyer D Sternbach M Spurll G. Hemolytic anemia following intravenous gamma globulin administration. Am J Med (1987) 82:633–5. 10.1016/0002-9343(87)90112-4 3826125

[107] Copelan EA Strohm PL Kennedy MS Tutschka PJ. Hemolysis following intravenous immune globulin therapy. Transfusion (1986) 26:410–2. 10.1046/j.1537-2995.1986.26587020113.x 3765031

[108] Atrah HI Crawford RJ Templeton JG Carlyle JE Transient haemoglobin drop following high dose intravenous immunoglobulin. Clin Lab Haematol (1985) 7:283 10.1111/j.1365-2257.1985.tb00038.x 4075743

[109] Kessary-Shoham H Levy Y Shoenfeld Y Lorber M Gershon H. *In vivo* administration of intravenous immunoglobulin (IVIg) can lead to enhanced erythrocyte sequestration. J Autoimmun (1999) 13:129–35. 10.1006/jaut.1999.0302 10441177

[110] Quinti I Pulvirenti F Milito C Granata G La Marra F Farrugia A Hemolysis in patients with antibody deficiencies on immunoglobulin replacement treatment. Transfusion (2014). 10.1111/trf.12939 25532440

[111] Thorpe SJ Fox BJ Dolman CD Lawrence J Thorpe R. Batches of intravenous immunoglobulin associated with adverse reactions in recipients contain atypically high anti-Rh D activity. Vox Sang (2003) 85:80–4. 10.1046/j.1423-0410.2003.00336.x 12925158

[112] Baxley A Akhtari M. Hematologic toxicities associated with intravenous immunoglobulin therapy. Int Immunopharmacol (2011) 11:1663–7. 10.1016/j.intimp.2011.07.024 21843660

[113] Thorpe SJ Fox BJ Dolman CD Thorpe R. Anti-A and anti-B activity in batches of different intravenous immunoglobulin products determined using a direct haemagglutination method. Biologicals (2005) 33:111–6. 10.1016/j.biologicals.2005.02.002 15905100

[114] Thorpe SJ Fox B Sharp G Virata ML Yu MW Thorpe R. International collaborative study to evaluate candidate reference reagents to standardize haemagglutination testing for anti-A and anti-B in normal intravenous immunoglobulin products. Vox Sang (2009) 97:160–8. 10.1111/j.1423-0410.2009.01194.x 19402856

[115] European Directorate for the Quality of Medicines and Healthcare. Human Normal Immunoglobulin for Intravenous Administration. 7th Monograph 0918 ed European Pharmacopoeia. Strasbourg: Council of Europe (2011). p. 4166–8.

[116] European Directorate for the Quality of Medicines and Healthcare. Anti-A and Anti-B Haemagglutinins. 7th General Chapter 2.6.20 ed European Pharmacopoeia. Strasbourg: Council of Europe (2011).

[117] European Directorate for the Quality of Medicines and Healthcare. Test for Anti-D Antibodies in Human Immunoglobulin. 7th General Chapter 2.6.26 ed European Pharmacopoeia. Strasbourg: Council of Europe (2011). 3546 p.

[118] Shoham-Kessary H Naot Y Gershon H. Immune complex-like moieties in immunoglobulin for intravenous use (i.v.Ig) bind complement and enhance phagocytosis of human erythrocytes. Clin Exp Immunol (1998) 113:77–84. 10.1046/j.1365-2249.1998.00624.x 9697987 PMC1905024

[119] Hudson KE Lin E Hendrickson JE Lukacher AE Zimring JC. Regulation of primary alloantibody response through antecedent exposure to a microbial T-cell epitope. Blood (2010) 115:3989–96. 10.1182/blood-2009-08-238568 20086249 PMC2869558

[120] Rodeghiero F Schiavotto C Castaman G Vespignani M Ruggeri M Dini E. A follow-up study of 49 adult patients with idiopathic thrombocytopenic purpura treated with high-dose immunoglobulins and anti-D immunoglobulins. Haematologica (1992) 77:248–52. 1330848

[121] Padmore RF. Hemolysis upon intravenous immunoglobulin transfusion. Transfus Apher Sci (2012) 46:93–6. 10.1016/j.transci.2011.11.004 22169381

[122] Report of the FDA meeting on Strategies to address Hemolytic complications of Immune Globulin Infusions. Washington, DC: US FDA (2014).

[123] Sewell WA Kerr J Behr-Gross ME Peter HH Kreuth Ig Working Group. European consensus proposal for immunoglobulin therapies. Eur J Immunol (2014) 44:2207–14. 10.1002/eji.201444700 24975475

[124] Jacobson NM The art of balanced production. In: Valverde JL, editor. Pharmaceuticals Policy and Law – Blood, Plasma and Plasma Proteins: A Unique Contribution to Modern Healthcare (Vol. 7), Amsterdam, NL: IOS Press (2006). p. 81–7.

[125] Dhainaut F Guillaumat PO Dib H Perret G Sauger A De Coupade C In vitro and in vivo properties differ among liquid intravenous immunoglobulin preparations. Vox Sang (2013) 104:115–26. 10.1111/j.1423-0410.2012.01648.x 23003576 PMC3580880

[126] Roch B Monrepos S Dhainaut F Milchaski C De Coupade C Sauger A Patient safety through an IVIg mastered manufacturing process. Posters. European Symposium Proceedings European Directorate for the Quality of Medicines & HealthCare (2013). p. 1–351 Available from: https://www.edqm.eu/medias/fichiers/posters_kreuth_iii.pdf

[127] Siani B Willimann K Wymann S Marques AA Widmer E. Isoagglutinin reduction in human immunoglobulin products by donor screening. Biol Ther (2014) 4:15–26. 10.1007/s13554-014-0016-2 24841428 PMC4254866

[128] Höfferer L IgG product development isoagglutinin reduction measures. Oral presentation, International Plasma Protein Congress Vienna: Plasma Protein Therapeutics Association (2014).

[129] Späth PJ Lutz HU. Naturally occurring antibodies/autoantibodies in polyclonal immunoglobulin concentrates. Lutz, H. U. Naturally Occurring Antibodies (NAbs). Adv Exp Med Biol (2012) 750:239–61. 10.1007/978-1-4614-3461-0_18 22903679

[130] Lutz HU Bussolino BF Flepp R Fasler S Stammler P Kazatchkine MD Naturally occurring anti-band-3 antibodies and complement together mediate phagocytosis of oxidatively stressed human erythrocytes. Proc Natl Acad Sci USA (1987) 84:7368–72. 10.1073/pnas.84.21.7368 3313392 PMC299297

[131] Lutz HU Stammler P Jelezarova E Nater M Späth PJ. High doses of immunoglobulin G attenuate immune aggregate-mediated complement activation by enhancing physiologic cleavage of C3b in C3bn-IgG complexes. Blood (1996) 88:184–93. 8704173

[132] Lutz HU Stammler P Bianchi V Trüeb RM Hunziker T Burger R. Intravenously applied IgG stimulates complement attenuation in a complement-dependent autoimmune disease at the amplifying C3 convertase level. Blood (2004) 103:465–72. 10.1182/blood-2003-05-1530 14512320

[133] Nydegger UE Tevaearai H Berdat P Rieben R Carrel T Mohacsi P Histo-blood group antigens as allo- and autoantigens. Ann N Y Acad Sci (2005) 1050:40–51. 10.1196/annals.1313.006 16014519

[134] Springer GF Horton RE. Blood group isoantibody stimulation in man by feeding blood group-active bacteria. J Clin Invest (1969) 48:1280–91. 10.1172/JCI106094 4893685 PMC322351

[135] Spalter SH Kaveri SV Bonnin E Mani JC Cartron JP Kazatchkine MD. Normal human serum contains natural antibodies reactive with autologous AB0 blood group antigens. Blood (1999) 93:4418–24. 10361140

[136] Galili U Rachmilewitz EA Peleg A Flechner I. A unique natural human IgG antibody with anti-alpha-galactosyl specificity. J Exp Med (1984) 160:1519–31. 10.1084/jem.160.5.1519 6491603 PMC2187506

[137] Obukhova P Rieben R Bovin N. Normal human serum contains high levels of anti-Galα1-4GlcNAc antibodies. Xenotransplantation (2007) 14:627–35. 10.1111/j.1399-3089.2007.00436.x 17991151

[138] Obukhova P Korchagina E Henry S Bovin N. Natural anti-A and anti-B of the AB0 system: allo- and autoantibodies have different epitope specificity. Transfusion (2012) 52:860–9. 10.1111/j.1537-2995.2011.03381.x 21981750

[139] Bovin N Bovin N Obukhova P Shilova N Rapoport E Popova I Repertoire of human natural anti-glycan immunoglobulins. Do we have auto-antibodies? Biochim Biophys Acta (2012) 1820:1373–82. 10.1016/j.bbagen.2012.02.005 22365885

[140] Galili U Mandrell RE Hamadeh RM Shohet SB McLeod Griffiss J. Interaction between human natural anti-alpha-galactosyl immunoglobulin G and bacteria of the human flora. Infect Immun (1988) 56:1730–7. 3290105 10.1128/iai.56.7.1730-1737.1988 PMC259469

[141] Lutz HU. Innate immune and non-immune mediators of erythrocyte clearance. Cell Mol Biol (Noisy-le-Grand) (2004) 50:107–16. 15095782

[142] Sorette MP Galili U Clark MR. Comparison of serum anti-band 3 and anti-Gal antibody binding to density-separated human red blood cells. Blood (1991) 77:628–36. 1991171

[143] Wieslander J Mansson O Kallin E Gabrielli A Nowack H Timple R Specificity of human antibodies against galα1-3gal carbohydrate epitope and distinction from natural antibodies reacting with galα1-2gal or galα1-4 gal. Glycoconj J (1990) 7:85–100 10.1007/BF01050405

[144] Nardini C Anti-A and anti-B haemagglutinin trend analysis during manufacturing process of IVIG. Presentation at Workshop “Strategies to Address Hemolytic Complications of Immune Globulin Infusions” Washington, DC: US FDA (2014).

[145] Rieben R Korchagina EY von Allmen E Hovinga JK Lämmle B Jungi TW. In vitro evaluation of the efficacy and biocompatibility of new, synthetic AB0 immunoabsorbents. Transplantation (1995) 60:425–30. 10.1097/00007890-199509000-00004 7676488

[146] Alikhani A Korchagina EY Chinarev AA Bovin NV Federspiel WJ. High molecular weight blood group A trisaccharide-polyacrylamide glycoconjugates as synthetic blood group A antigens for anti-A antibody removal devices. J Biomed Mater Res B Appl Biomater (2009) 91:845–54. 10.1002/jbm.b.31466 19582848 PMC5944835

[147] Gautam S Korchagina EY Bovin NV Federspiel WJ. Specific antibody filter (SAF) binding capacity enhancement to remove anti-A antibodies. J Biomed Mater Res B Appl Biomater (2010) 95:475–80. 10.1002/jbm.b.31707 20878917

[148] Orbach H Katz U Sherer Y Shoenfeld Y. Intravenous immunoglobulin: adverse effects and safe administration. Clin Rev Allergy Immunol (2005) 29:173–84. 10.1385/CRIAI:29:3:173 16391392

[149] Rajabally YA Kearney DA. Thromboembolic complications of intravenous immunoglobulin therapy in patients with neuropathy: a two-year study. J Neurol Sci (2011) 308:124–7. 10.1016/j.jns.2011.05.035 21679973

[150] Dalakas MC. High-dose intravenous immunoglobulin and serum viscosity: risk of precipitating thromboembolic events. Neurology (1994) 44:223–6. 10.1212/WNL.44.2.223 8309562

[151] Sakem B Matozan K Nydegger UE Weigel G Griesmacher A Risch L. Anti-red blood cell antibodies, free light chains, and antiphospholipid antibodies in intravenous immunoglobulin preparations. Isr Med Assoc J (2013) 15:617–21. 24266088

[152] Grosse-Wilde H Blasczyk R Westhoff U. Soluble HLA class I and class II concentrations in commercial immunoglobulin preparations. Tissue Antigens (1992) 39:74–7. 10.1111/j.1399-0039.1992.tb01910.x 1574801

[153] Sztajzel R Le Floch-Rohr J Eggimann P. High-dose intravenous immunoglobulin treatment and cerebral vasospasm: a possible mechanism of ischemic encephalopathy? Eur Neurol (1999) 41:153–8. 10.1159/000008040 10202247

[154] Vinod KV Kumar M Nisar KK. High dose intravenous immunoglobulin may be complicated by myocardial infarction. Indian J Crit Care Med (2014) 18:247–9. 10.4103/0972-5229.130579 24872657 PMC4033861

[155] Iroh Tam PY Richardson M Grewal S. Fatal case of bilateral internal jugular vein thrombosis following IVIg infusion in an adolescent girl treated for ITP. Am J Hematol (2008) 83:323–5. 10.1002/ajh.21107 17975805

[156] Al-Riyami AZ Lee J Connolly M Shereck E. Cerebral sinus thrombosis following IV immunoglobulin therapy of immune thrombocytopenia purpura. Pediatr Blood Cancer (2011) 57:157–9. 10.1002/pbc.22968 21445949

[157] Sin YH Kim YJ Oh JS Lee JH Kim SM Kim JK. Graft rupture after high-dose intravenous immunoglobulin therapy in a renal transplant patient. Nephrology (2014) 19:35–6. 10.1111/nep.12248 24842820

[158] Barada W Muwakkit S Hourani R Bitar M Mikati M. Cerebral sinus thrombosis in a patient with humoral immunodeficiency on intravenous immunoglobulin therapy: a case report. Neuropediatrics (2008) 39:131–3. 10.1055/s-2008-1077088 18671192

[159] Lee YJ Shin JU Lee J Kim K Kim WS Ahn JS A case of deep vein thrombosis and pulmonary thromboembolism after intravenous immunoglobulin therapy. J Korean Med Sci (2007) 22:758–61. 10.3346/jkms.2007.22.4.758 17728525 PMC2693835

[160] White DA Leonard MC. Acute stroke with high-dose intravenous immune globulin. Am J Health Syst Pharm (2007) 64:1611–4. 10.2146/ajhp060205 17646563

[161] Feuillet L Milandre L Ali Cherif A Venous and arterial thrombosis following administration of intravenous immunoglobulins. Blood Coagul Fibrinolysis (2006) 17:85 10.1097/01.mbc.0000194367.37721.a5 16607087

[162] Marie I Hervé F Kerleau JM Maurey G Levesque H Intravenous immunoglobulin-associated vena cava thrombosis. Thromb Haemost (2006) 96:849–51 10.1160/TH06-09-0503 17139383

[163] Marie I Maurey G Hervé F Hellot MF Levesque H. Intravenous immunoglobulin-associated arterial and venous thrombosis; report of a series and review of the literature. Br J Dermatol (2006) 155:714–21. 10.1111/j.1365-2133.2006.07390.x 16965420

[164] Hefer D Jaloudi M Thromboembolic events as an emerging adverse effect during high-dose intravenous immunoglobulin therapy in elderly patients: a case report and discussion of the relevant literature. Ann Hematol (2004) 83:661–5 10.1007/s00277-004-0895-2 15309520

[165] Geller JL Hackner D. Diffuse venous thromboemboli associated with IVIg therapy in the treatment of streptococcal toxic shock syndrome: case report and review. Ann Hematol (2005) 84:601–4. 10.1007/s00277-005-1043-3 15815906

[166] Feuillet L Guedj E Laksiri N Philip E Habib G Pelletier J Deep vein thrombosis after intravenous immunoglobulins associated with methylprednisolone. Thromb Haemost (2004) 92:662–5. 15457603

[167] Sheehan DJ Lesher JL. Deep venous thrombosis after high-dose intravenous immunoglobulin in the treatment of pemphigus vulgaris. Cutis (2004) 73:403–6. 15224785

[168] Vucic S Chong PS Dawson KT Cudkowicz M Cros D Jr Thromboembolic complications of intravenous immunoglobulin treatment. Eur Neurol (2004) 52:141–4 10.1159/000081465 15479982

[169] Stamboulis E Theodorou V Kilidireas K Apostolou T Acute myocardial infarction following intravenous immunoglobulin therapy for chronic inflammatory demyelinating polyneuropathy in association with a monoclonal immunoglobulin G paraprotein. Eur Neurol (2004) 51:51 10.1159/000075091 14639034

[170] Katz KA Hivnor CM Geist DE Shapiro M Ming ME Werth VP Stroke and deep venous thrombosis complicating intravenous immunoglobulin infusions. Arch Dermatol (2003) 139:991–3 10.1001/archderm.139.8.991 12925384

[171] Zaidan R Al Moallem M Wani BA Shameena AR Al Tahan AR Daif AK Thrombosis complicating high dose intravenous immunoglobulin: report of three cases and review of the literature. Eur J Neurol (2003) 10:367–72. 10.1046/j.1468-1331.2003.00542.x 12823487

[172] Brown HC Ballas ZK Acute thromboembolic events associated with intravenous immunoglobulin infusion in antibody-deficient patients. J Allergy Clin Immunol (2003) 112:797–9 10.1016/S0091-6749(03)01780-9 14564366

[173] Evangelou N Littlewood T Anslow P Chapel H. Transverse sinus thrombosis and IVIg treatment: a case report and discussion of risk-benefit assessment for immunoglobulin treatment. J Clin Pathol (2003) 56:308–9. 10.1136/jcp.56.4.308 12663646 PMC1769931

[174] Emerson GG Herndon CN Sreih AG. Thrombotic complications after intravenous immunoglobulin therapy in two patients. Pharmacotherapy (2002) 22:1638–41. 10.1592/phco.22.17.1638.34125 12495174

[175] Alliot C Rapin JP Besson M Bedjaoui F Messouak D Pulmonary embolism after intravenous immunoglobulin. J R Soc Med (2001) 94:187–8. 11317625 10.1177/014107680109400412 PMC1281394

[176] Sherer Y Levy Y Langevitz P Rauova L Fabrizzi F Shoenfeld Y Adverse effects of intravenous immunoglobulin therapy in 56 patients with autoimmune diseases. Pharmacology (2001) 62:133–7 10.1159/000056085 11287813

[177] Elkayam O Paran D Milo R Davidovitz Y Almoznino-Sarafian D Zeltser D Acute myocardial infarction associated with high dose intravenous immunoglobulin infusion for autoimmune disorders. A study of four cases. Ann Rheum Dis (2000) 59:77–80. 10.1136/ard.59.1.77 10627434 PMC1752991

[178] Go RS Call TG. Deep venous thrombosis of the arm after intravenous immunoglobulin infusion: case report and literature review of intravenous immunoglobulin-related thrombotic complications. Mayo Clin Proc (2000) 75:83–5. 10.4065/75.1.83 10630762

[179] Turner B Wills AJ. Cerebral infarction complicating intravenous immunoglobulin therapy in a patient with Miller Fisher syndrome. J Neurol Neurosurg Psychiatry (2000) 68:790–1. 10.1136/jnnp.68.6.790 10811710 PMC1736949

[180] Harkness KA Goulding P Central retinal vein occlusion complicating treatment with intravenous immunoglobulin. Eye (Lond) (2000) 14:662–3 10.1038/eye.2000.163 11040920

[181] Paolini R Fabris F Cella G Acute myocardial infarction during treatment with intravenous immunoglobulin for idiopathic thrombocytopenic purpura (ITP). Am J Hematol (2000) 65:177–8 10.1002/1096-8652(200010)65:2<177::AID-AJH17>3.0.CO;2-K 10996840

[182] Rosenbaum JT Myocardial infarction as a complication of immunoglobulin therapy. Arthritis Rheum (1997) 40:1732–3 10.1002/art.38 9324038

[183] Oh KT Boldt HC Danis RP. Iatrogenic central retinal vein occlusion and hyperviscosity associated with high-dose intravenous immunoglobulin administration. Am J Ophthalmol (1997) 124:416–8. 10.1016/S0002-9394(14)70844-X 9439378

